# Advances in Periodontal Diagnostics: Application of MultiModal Language Models in Visual Interpretation of Panoramic Radiographs

**DOI:** 10.3390/diagnostics15151851

**Published:** 2025-07-23

**Authors:** Albert Camlet, Aida Kusiak, Agata Ossowska, Dariusz Świetlik

**Affiliations:** 1 Department of Periodontology and Oral Mucosa Diseases, Medical University of Gdansk, Orzeszkowej 18 St., 80-208 Gdansk, Poland; acamlet@gumed.edu.pl (A.C.); akusiak@gumed.edu.pl (A.K.); agata.ossowska@gumed.edu.pl (A.O.); 2 Division of Biostatistics and Neural Networks, Medical University of Gdansk, Debinki 1 St., 80-211 Gdansk, Poland

**Keywords:** periodontitis, bone loss, panoramic radiographs, large language models, multimodal language models, ChatGPT, convolutional neural networks, deep learning

## Abstract

**Background**: Periodontitis is a multifactorial disease leading to the loss of clinical attachment and alveolar bone. The diagnosis of periodontitis involves a clinical examination and radiographic evaluation, including panoramic images. Panoramic radiographs are cost-effective methods widely used in periodontitis classification. The remaining bone height (RBH) is a parameter used to assess the alveolar bone level. Large language models are widely utilized in the medical sciences. ChatGPT, the leading conversational model, has recently been extended to process visual data. The aim of this study was to assess the effectiveness of the ChatGPT models 4.5, o1, o3 and o4-mini-high in RBH measurement and tooth counts in relation to dental professionals’ evaluations. **Methods**: The analysis was based on 10 panoramic images, from which 252, 251, 246 and 271 approximal sites were qualified for the RBH measurement (using the models 4.5, o1, o3 and o4-mini-high, respectively). Three examiners were asked to independently evaluate the RBH in approximal sites, while the tooth count was achieved by consensus. Subsequently, the results were compared with the ChatGPT outputs. **Results**: ChatGPT 4.5, ChatGPT o3 and ChatGPT o4-mini-high achieved substantial agreement with clinicians in the assessment of tooth counts (κ = 0.65, κ = 0.66, κ = 0.69, respectively), while ChatGPT o1 achieved moderate agreement (κ = 0.52). In the context of RBH values, the ChatGPT models consistently exhibited a positive mean bias compared with the clinicians. ChatGPT 4.5 was reported to provide the lowest bias (+12 percentage points (pp) for the distal surfaces, width of the 95% CI for limits of agreement (LoAs) ~60 pp; +11 pp for the mesial surfaces, LoA width ~54 pp). **Conclusions**: ChatGPT 4.5 and ChatGPT o3 show potential in the assessment of tooth counts on a panoramic radiograph; however, their present level of accuracy is insufficient for clinical use. In the current stage of development, the ChatGPT models substantially overestimated the RBH values; therefore, they are not applicable for classifying periodontal disease.

## 1. Introduction

Periodontitis is a multifactorial disease related to a dysbiotic biofilm. The disease is characterized by chronic inflammation of tooth-supporting structures, which leads to the destruction of both connective and bone tissues [1]. Periodontitis represents a major challenge to global health. The Global Burden of Disease Study 2021 demonstrated that 1.06 billion people worldwide are affected by severe periodontitis. Periodontitis was most frequently diagnosed in the age group 50–64 years. By the year 2050, the number of individuals with periodontitis may exceed 1.5 billion [2]. Numerous studies confirmed the association between periodontitis and other diseases, including diabetes, rheumatoid arthritis, cardiovascular diseases and respiratory diseases [3,4,5,6]. Agarwal et al. conducted a systematic review and meta-analysis in which they identified a significant association between tooth loss, periodontitis and mild cognitive impairment. The authors highlighted the importance of developing diagnostic and treatment strategies for patients who may develop oral diseases [7]. According to the 2017 World Workshop on the Classification of Periodontal and Peri-Implant Diseases and Conditions, periodontitis is recognized in the presence of clinical attachment loss (CAL), radiographic alveolar bone loss, pathological pockets and gingival bleeding. CAL is measured by probing the periodontal pocket, while bone loss is assessed using various radiographic imaging techniques. Both CAL and radiographic bone loss are measured at approximal sites of a tooth [8]. Radiographic bone loss is determined by the vertical distance between the cemento-enamel junction (CEJ) and the alveolar crest [9]. Another parameter described in the literature for assessing bone level is the RBH—remaining bone height [10].

In periodontology, a wide range of imaging modalities is applied, including two-dimensional (2D) and three-dimensional (3D) techniques. Periapical and bitewing radiographs are the representatives of 2D intraoral images, while panoramic dental radiographs are 2D extraoral images. Cone-beam computed tomography allows for a more precise reconstruction of the dentomaxillo-facial region across three orthogonal planes [11]. The diagnostic effectiveness of numerous imaging techniques has been widely explored in the academic literature. Panoramic imaging is recognized as a cost-effective diagnostic technique. Moreover, it facilitates a thorough assessment of the maxilla and mandible in a single, low-dose radiation exposure. However, panoramic radiography involves significant limitations, including the overlapping of anatomical structures, limited effectiveness in detecting minor pathologies and a distortion of proportions between certain areas [12,13].

Large language models are an advanced type of deep learning systems trained on an immense volume of textual data. LLMs are effectively utilized in tasks requiring natural language processing, for example, text generation, classification and language translation [14]. LLMs have emerged as supportive tools in various branches of medicine, particularly in radiology, orthopedics, dermatology, cardiology, pediatrics, neurology and public health [15,16,17,18,19,20,21]. LLMs are also widely utilized across dental specialties. LLMs could be successfully applied in oral and maxillofacial surgery for the development of informed consent forms, as well as in the postoperative follow-up evaluation [22]. ChatGPT is a large language model based on the Transformer architecture, a type of neural network designed for processing human-like text [23]. In the field of periodontology, ChatGPT may be utilized in periodontal disease classification, as well as a valuable source of information about periodontitis [24,25] and to support postgraduate education at the specialist level [26]. Although ChatGPT uses the transformer architecture—a deep neural network with a self-attention mechanism optimized for natural language processing—classical neural networks are also used to assess the severity of periodontitis, with a particular emphasis on clinical parameters and risk factors [27,28].

While the general idea of transformer models is to process textual data, the concept of ChatGPT is increasingly evolving and it is gaining new abilities. The recent versions of ChatGPT (versions 4.5, 4o, o1, o3, o4-mini-high) have obtained the functionality to analyze documents, photos, images and other forms of data. Currently, little is known about ChatGPT’s application in the visual interpretation of dental panoramic images, particularly in the assessment of alveolar bone level and bone loss. Considering the significant prevalence of periodontitis in the global population, it is notably important to search for effective and accessible diagnostic methods for general practice dentists. ChatGPT, the fastest growing conversational model, might fulfill the role of a relevant diagnostic tool in periodontal disease. The aims of this study were the following:

1. Evaluate the agreement in teeth identification between ChatGPT and dental professionals.
2. Evaluate the differences between measurements of remaining bone height (RBH) performed by ChatGPT models.
3. Evaluate the agreement in RBH values between ChatGPT and dental professionals.
4. Assess the clinical relevance of ChatGPT in dental radiology at its current stage of advancement.

## 2. Materials and Methods

### 2.1. Ethics Statement

This study was conducted in accordance with the Declaration of Helsinki and approved by the Institutional Review Board of Medical University of Gdansk (IRB No.: KB/517/2024) on 12 December 2024. Regarding the ethics committee’s approval and the retrospective character of the study, informed consent from patients was not applicable.

### 2.2. Definitions

Interproximal bone loss is described in various ways in the medical literature. According to the 2017 Classification of Periodontal and Peri-Implant Diseases and Conditions, “radiographic bone loss” is an officially recognized and used term [8]. Equivalent terms, including “periodontal bone loss”, “oral bone loss” and “alveolar bone loss”, are also found in the scientific literature [29,30,31,32,33]. The mentioned terms refer to the distance from the cemento-enamel junction (CEJ) to the alveolar bone crest (also known as the marginal bone crest) divided by the distance from the CEJ to the root apex and presented as a percentage value [34]. Moreover, in the literature, the “remaining bone height” is also applied and calculated using the same reference points. The concept of the radiological assessment of remaining bone height (RBH) was derived from original work by Ryden et al. [10]. The standardized protocol of measurement was successfully implemented in another study by Machado et al. [35]. The remaining bone height (RBH) is defined as the arithmetic value between two linear distances. The first measurement is obtained from the following reference points: marginal bone crest to the radiographic root apex. The second measurement is taken from the cemento-enamel junction to the radiographic root apex. The first parameter corresponds to the portion of the root supported by the alveolar bone, while the second parameter reflects the total root length. The RBH can be presented using the following formula:

$$RBH(\%)=\left(\frac{marginal\;bone\;crest\;-\;radiographic\;root\;apex}{cementoenamel\;juction\;-\;radiographic\;root\;apex}\right)\times 100$$

### 2.3. Source Data and Eligibility Criteria

Panoramic images were obtained by a certified technician with the use of Carestream CS 9300 C (Carestream Dental LLC, Atlanta, GA, USA) at the University Dental Centre. The radiographic images were obtained directly from the internal electronic database in their original form. No part of the panoramic images carried personal information. The exposure parameters were within the ranges of 68–78 kVp and 8–12 mA. The exposure times varied from 13.5 to 15.2 s. The original pixel resolution of the images was 1920 × 930.

One hundred panoramic images were randomly selected from the electronic database of University Dental Centre and underwent an eligibility assessment. The inclusion and exclusion criteria have been established by the consensus of three examiners that attended the study. The exclusion process was divided into two steps: the first step related to the general evaluation of panoramic images. The second step was focused on rejecting the specific approximal surfaces according to the exclusion criteria.

Inclusion criteria for panoramic radiographs:

1. Panoramic images obtained from adult patients treated for periodontitis at the Department of Periodontal and Oral Mucosa Diseases between 2018 and 2024.

Exclusion criteria for panoramic radiographs:

1. Patients with less than 4 teeth;
2. Poor quality of the panoramic image;
3. Severely blurred image due to patient movement;
4. Extensive fixed prosthetic appliances, including multiple-unit dental bridges;
5. Complex malocclusions associated with teeth crowding;
6. Patients who had received radiotherapy or resective surgery;
7. Patients with abnormalities of the maxillofacial region.

Exclusion criteria for the approximal surfaces:

1. Disturbed visibility of the cemento-enamel junction due to caries, massive restorations and teeth rotation;
2. Disturbed visibility of the root apex due to the overlapping of anatomical structures and poor quality of the area covered;
3. Disturbed visibility of the marginal bone crest due to the overlapping of anatomical structures.

**Figure 1:**
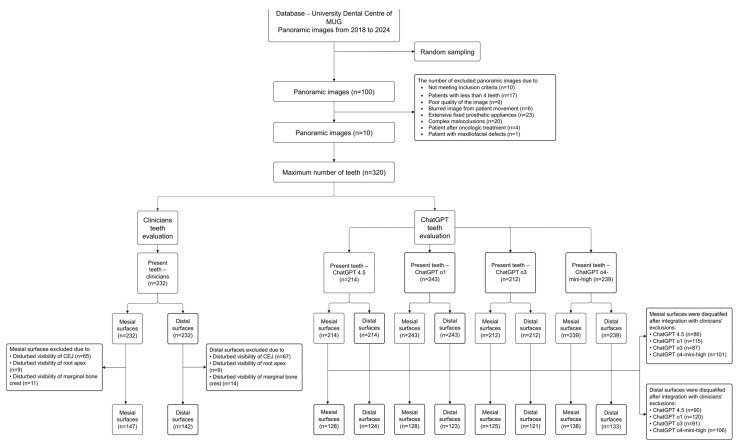
Flow diagram of data inclusion and exclusion.

Figure 1 in the upper section presents information regarding the source from which the pre-screening dataset of panoramic images was retrieved, along with the exclusion criteria and the corresponding number of images rejected. The lower section presents information on the number of teeth identified by the clinicians and the ChatGPT models, as well as the final number of approximal surfaces included in the study following the second stage of exclusion.

### 2.4. Tooth Count, Remaining Bone Height (RBH) Measurement and Data Collection

Regarding the eligibility criteria, a total of 10 panoramic radiographs were included in the study. The analysis of panoramic images focused on two main parameters: the number of present teeth and the remaining bone height (RBH) for each approximal surface (mesial and distal). The radiographs were examined by clinicians under reproducible conditions, as well as by ChatGPT models guided by a uniform textual instruction. Subsequently, the results were collected and transferred to statistical analysis.

#### 2.4.1. Analysis Conducted by Clinicians

**Figure 2:**
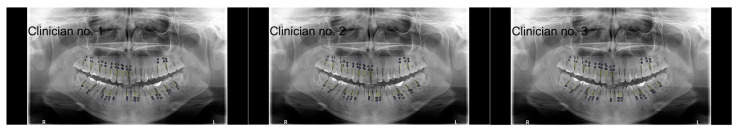
The panoramic radiograph with measurement lines marked by three independent clinicians.

The analysis was conducted in a room with controlled lighting conditions. The images were reviewed on a high-resolution monitor. The presence of specific teeth was established by a consensus. Regarding the RBH values, the examiners independently analyzed the panoramic images with the use of image analysis software ImageJ (version 1.54 g; National Institutes of Health, Bethesda, MD, USA). The software is available in the public domain. Initially, the reference points were identified on the images. Based on the measured lines (the blue line refers to the distance between the marginal bone crest and the radiographic root apex, while the yellow line refers to the distance between the cemento-enamel junction and the radiographic root apex), the RBH was calculated individually for each tooth on the mesial and distal surfaces. The results were exported to a spreadsheet for the statistical analysis. Figure 2 shows the example of a single panoramic radiograph repeated three times and analyzed by three independent examiners. Each image contains annotated measurement lines that are visually distinguished by color (blue and yellow lines).

#### 2.4.2. Analysis Conducted by ChatGPT

**Figure 3:**
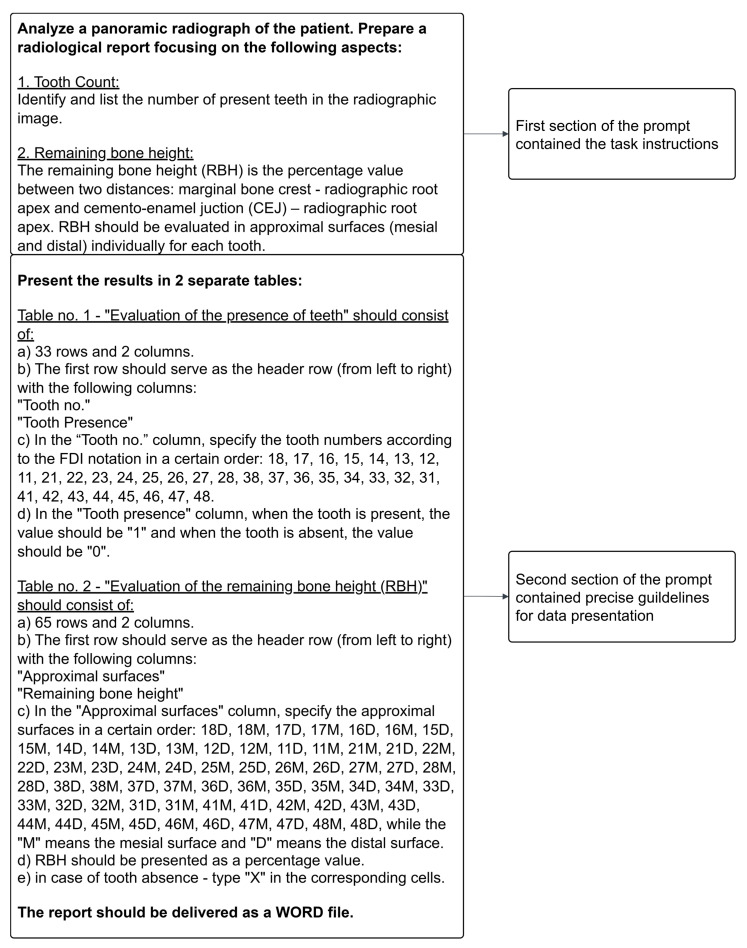
Instructions for ChatGPT models.

The research was conducted with the use of ChatGPT 4.5 (released on 27 February 2025), ChatGPT o1 (released on 12 September 2024), ChatGPT o3 and o4-mini-high (released on 16 April 2025; OpenAI Inc., San Francisco, CA, USA). A distinctive feature of multimodal ChatGPT models is their ability to integrate text data with images. Moreover, transformer models do not require training on strictly labeled data. ChatGPT models are pre-trained on large-scale datasets. Consequently, in our study, their capability to process panoramic radiographs was evaluated solely based on textual instructions. The models had not been specifically trained on panoramic radiographs prior to the analysis. ChatGPT allows users to enter prompts in the “Project” mode, enabling the standardized application of instructions to each input. This mode eliminates the necessity for the manual insertion of instructions each time. The instructions used in the study were divided into two sections. The first section specified the investigated parameters: the presence of teeth and remaining bone height (RBH). The second section provided precise guidelines for the structured data presentation (Figure 3). Panoramic images were individually uploaded as PNG files via a dedicated input box. The results were collected in two ways, depending on how the data were presented by ChatGPT: by direct download of a generated report in Docx format or by manual transfer into a specified text file. The reports are available in the “Supplementary Materials”.

Figure 3 presents the structure of the prompt, which was used to interact with ChatGPT. The first section contained detailed instructions on the data ChatGPT was expected to find: the presence of teeth and remaining bone height (RBH) for the approximal surface. The second section provided a detailed description of the data presentation method: two tables with a defined number of rows, columns and headers. Additionally, specific instructions were included to standardize the presentation of the results: the presence of a tooth was indicated by the value “1”, while the absence was marked by “0”. The remaining bone height (RBH) values were expressed as percentages; however, in cases of a tooth missing (along with the corresponding approximal surfaces), the value “X” was assigned.

### 2.5. Statistical Analysis

The statistical analyses were performed with Statistica (TIBCO Software Inc., San Ramon, CA, USA; version 13, 2017) (https://www.tibco.com/, accessed on 27 November 2023). The statistical analyses were conducted using a significance level of 0.05 and all estimates are reported with their corresponding 95% confidence intervals (95% CIs). Agreement between categorical assessments was quantified using Cohen’s kappa coefficients, with interpretations based on standard guidelines. Continuous measurement agreement was further evaluated via a Bland–Altman analysis that assessed the mean bias and limits of agreement. The reliability of the quantitative variables was determined using the intraclass correlation coefficient (ICC) calculated under a two-way random-effects model. Group comparisons for the non-normally distributed data were performed using the Kruskal–Wallis test, followed by post hoc pairwise comparisons with a Bonferroni correction. 

## 3. Results

### 3.1. Number of Teeth Evaluated by Clinicians and ChatGPT Models

The total number of teeth identified by the clinicians on the 10 panoramic images was determined to be 232 (72.5% of the maximum number of teeth). ChatGPT 4.5, o1, o3 and o4-mini-high identified 214, 243, 212 and 239 teeth, respectively. The agreement between the clinicians and the ChatGPT 4.5, o3 and o4-mini-high models was reported to be substantial (κ = 0.65, 95% CI: 0.56–0.73; κ = 0.66, 95% CI: 0.57–0.75; κ = 0.69, 95% CI: 0.59–0.78, respectively). The Fleiss’ kappa coefficient for the clinicians–ChatGPT o1 pair was 0.52 (95% CI: 0.41–0.62), indicating a moderate agreement. Each of the models demonstrated a high sensitivity exceeding 85%, while representing different levels of specificity. ChatGPT o1 and ChatGPT o4-mini-high achieved lower levels of specificity compared with ChatGPT 4.5 and ChatGPT o3 (60.2%, 73.9% vs. 83.0%, 85.2%, respectively). The positive predictive value (PPV) was identified to be above 90% for ChatGPT 4.5, o3 and o4-mini. For ChatGPT o1, approximately 14 out of 100 indications were reported to be false positives (PPV = 85.6%). See Table 1.

### 3.2. Intraclass Correlation Coefficient for Remaining Bone Height (RBH) Values Between Clinicians

The intraclass correlation coefficient (ICC) between clinicians was measured as 0.96 for the distal surfaces (single-rater, 95% CI: 0.94–0.97) and 0.95 for the mesial surfaces (single-rater; 95% CI: 0.93–0.96). Regarding the average for three raters, the ICC was 0.99 for the distal surfaces (95% CI: 0.98–0.99) and 0.98 for the mesial surfaces (95% CI: 0.98–0.99). The parameters show an excellent agreement of the RBH measurements across the clinicians. See Table 2.

### 3.3. Summary of RBH Values for Distal and Mesial Surfaces Evaluated by Clinicians

Clinician 1 reported a mean RBH for the distal surfaces of 70.4% (SD = 11.6%; range = 28.0–85.0%; median = 72.0%; IQR = 16.0%; 95% CI = [66.2%; 74.6%]) and mesial surfaces of 68.8% (SD = 9.1%; range = 52.0–85.0%; median = 69.0%; IQR = 14.0%; 95% CI = [65.5%; 72.1%]). Clinician 2 obtained a mean RBH for the distal surfaces of 72.6% (SD = 12.4%; range = 31.0–86.0%; median = 75.0%; IQR = 18.5%; 95% CI = [68.2%; 77.1%]) and mesial surfaces of 70.7% (SD = 9.5%; range = 56.0–87.0%; median = 73.0%; IQR = 16.0%; 95% CI = [67.2%; 74.2%]). Clinician 3 achieved a mean RBH for the distal surfaces of 71.9% (SD = 12.8%; range = 26.0–89.0%; median = 75.5%; IQR = 15.0%; 95% CI = [67.3%; 76.5%]) and mesial surfaces of 69.7% (SD = 9.2%; range = 54.0–90.0%; median = 71.0%; IQR = 12.0%; 95% CI = [66.4%; 73.1%]). There were no statistically significant differences between the mean values of the RBH for the approximal surfaces (distal and mesial) between clinicians (*p* = 0.7853, *p* = 0.7933, respectively). See Table 3 and Table 4.

### 3.4. Summary of RBH Values for Distal and Mesial Surfaces Evaluated by ChatGPT Models

The analysis was restricted to the approximal surfaces of teeth that were accurately identified by both the clinicians and ChatGPT models and met the eligibility criteria. The number of included approximal surfaces were 128, 128, 125, and 138 (mesial surfaces) and 124, 123, 121, and 133 (distal surfaces) for ChatGPT 4.5, ChatGPT o1, ChatGPT o3 and ChatGPT o4-mini-high, respectively. ChatGPT 4.5 yielded a mean RBH for the distal surfaces of 73.7% (SD = 6.9%; range = 60.0–85.0%; median = 74.0%; IQR = 10.0%; 95% CI = [70.9%; 76.6%]) and mesial surfaces of 74.2% (SD = 6.8%; range = 55.0–85.0%; median = 75.0%; IQR = 10.0%; 95% CI = [71.4%; 77.1%]). ChatGPT o1 produced a mean RBH for the distal surfaces of 76.7% (SD = 15.4%; range = 40.0–90.0%; median = 85.0%; IQR = 20.0%; 95% CI = [69.6%; 83.7%]) and mesial surfaces of 75.8% (SD = 16.3%; range = 35.0–90.0%; median = 85.0%; IQR = 25.0%; 95% CI = [68.1%; 83.4%]). ChatGPT o3 yielded a mean RBH for the distal surfaces of 73.0% (SD = 12.4%; range = 60.0–92.0%; median = 70.0%; IQR = 25.0%; 95% CI = [67.6%; 78.4%]) and mesial surfaces of 74.2% (SD = 12.4%; range = 60.0–92.0%; median = 70.0%; IQR = 25.0%; 95% CI = [68.9%; 79.6%]). ChatGPT o4-mini-high produced a mean RBH for the distal surfaces of 77.0% (SD = 10.4%; range = 50.0–90.0%; median = 80.0%; IQR = 5.0%; 95% CI = [72.9%; 81.2%]) and mesial surfaces of 78.2% (SD = 10.5%; range = 45.0–92.0%; median = 80.0%; IQR = 0.0%; 95% CI = [74.0%; 82.5%]). There were statistically significant differences in the RBH measurements for the distal and mesial surfaces across different versions of ChatGPT: between versions o1 and o3, the *p*-values were 0.0053 and 0.0002, respectively. For the remaining version-to-version pairs, the *p*-values were <0.0001. See Table 5 and Table 6.

### 3.5. Agreement Between Clinicians and ChatGPT Models in RBH Measurement for Approximal Surfaces

**Figure 4:**
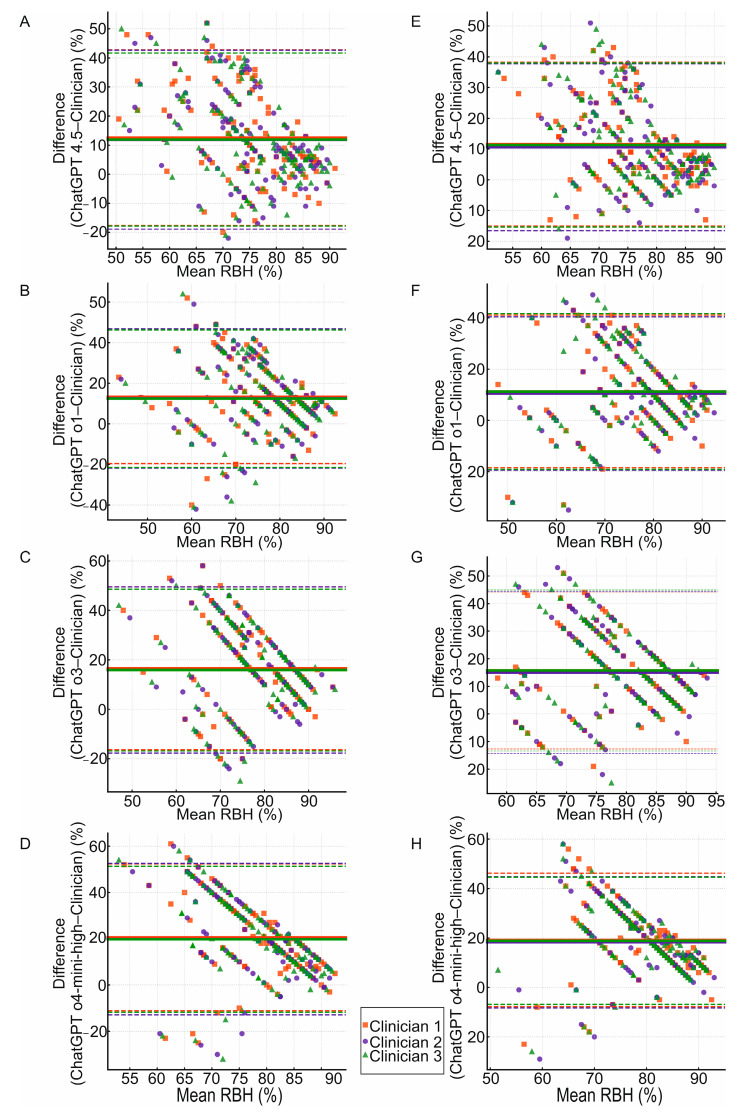
Bland–Altman plots illustrating the agreement of RBH assessment between the three clinicians and the ChatGPT models: (**A**) ChatGPT 4.5 at distal surfaces, (**B**) ChatGPT o1 at distal surfaces, (**C**) ChatGPT o3 at distal surfaces, (**D**) ChatGPT o4-mini-high at distal surfaces, (**E**) ChatGPT 4.5 at mesial surfaces, (**F**) ChatGPT o1 at mesial surfaces, (**G**) ChatGPT o3 at mesial surfaces, (**H**) ChatGPT o4-mini-high at mesial surfaces.

The Bland–Altman plot was utilized to search for agreement in the RBH measurements between ChatGPT and clinicians. Figure 4 presents eight Bland–Altman plots evaluating the agreement in the RBH assessment between various versions of ChatGPT and three clinicians. Plots A, B, C and D refer to the RBH at distal surfaces, while plots E, F, G and H refer to the RBH for mesial surfaces. The *X*-axis represents the mean percentage value of RBH measurements performed by the ChatGPT model and the clinician, while the *Y*-axis shows the difference between the two measurements. The multicolored solid horizontal line represents the mean bias, while the two multicolored dashed horizontal lines positioned peripherally indicate the limits of agreement. The lowest mean bias (95% CI) was observed between ChatGPT 4.5 and the three clinicians (for the distal surfaces: +13 percentage points (pp), +12 pp and +12 pp, respectively; for the mesial surfaces: +12 pp, +11 pp and +11 pp, respectively). The mean biases for ChatGPT o1 were observed to be +13 pp, +13 pp and +12 pp (for the distal surfaces) and a consistent +11 pp (for the mesial surfaces). ChatGPT o3 yielded mean biases of +17 pp, +16 pp and +16 pp (for the distal surfaces) and +16 pp, +15 pp and +16 pp (for the mesial surfaces). ChatGPT o4-mini-high was reported to yield the highest mean biases of +21 pp, +20 pp and +20 pp (for the distal surfaces) and +19 pp, +18 pp and +19 pp (for the mesial surfaces). All ChatGPT models presented wide limits of agreement (LoAs). See Table 7 and Table 8.

### 3.6. Comparison Between ChatGPT Models in RBH Measurement for Approximal Surfaces

Regarding the distal and mesial surfaces, a systematic positive bias was observed for each version. ChatGPT 4.5 was reported to generate the lowest mean bias of +12 pp for the distal surfaces. ChatGPT 4.5 and ChatGPT o1 jointly exhibited the lowest mean bias of +11 pp for the mesial surfaces. See Table 9 and Table 10.

## 4. Discussion

Numerous studies have addressed the dilemma of whether panoramic imaging is sufficient to assess alveolar bone levels, particularly in relation to other imaging modalities. Periapical radiography is frequently utilized in marginal bone loss. The long-cone paralleling technique provides reproducibility and comparative evaluation of the images [36]. Akesson found that periapical images were more precise in bone loss evaluation in comparison with bitewing and panoramic radiographs. However, the author emphasized that all the imaging methods tended to underestimate bone loss relative to clinical probing [37]. Conversely, previous research by Gedik et al. showed that bitewing radiographs provided the most reliable assessment of the bone level, while periapical radiographs demonstrated the lowest level of diagnostic accuracy [38]. Similarly, Manja and Fransiari demonstrated the highest effectiveness of bitewing radiographs in the bone loss evaluation in their study. However, there were no statistically significant differences between the periapical, panoramic and bitewing images [39]. According to Kim et al. panoramic radiography presents reasonable effectiveness for the initial assessment of bone loss. In cases of significant bone level changes, intraoral radiography is advised [40]. Cone-beam computed tomography (CBCT) is an imaging technique that allows for three-dimensional reconstruction. CBCT is widely applied in various fields of dentistry, including advanced implantology planning, orthodontics, endodontics, forensic dentistry, and oral and maxillofacial surgeries [41]. In the study by Takeshita et al., CBCT achieved the most comparable results with clinical evaluation regarding alveolar bone loss assessment and outperformed 2D imaging methods [42]. Numerous publications underscore the superiority of CBCT over other imaging methods in the assessment of bone loss at non-proximal surfaces [43,44]. In cases of complex, three-dimensional pathologies involving furcation lesions and intrabony defects, CBCT provides precise pre-treatment evaluation [45]. Nevertheless, according to a consensus statement from the American Academy of Periodontology, clinical examination and two-dimensional full mouth radiography are considered a gold standard in periodontal diagnosis. Therefore, CBCT should not be regarded as a routine diagnostic method [46]. Despite the periapical radiography is perceived as a targeted diagnostic tool providing accuracy and repeatability, panoramic imaging has proven effectiveness in periodontology. Persson et al. found that measurements from intra-oral radiographs and panoramic images demonstrate substantial agreement. The authors concluded that panoramic images may serve as a parallel diagnostic tool to intraoral radiography for alveolar bone loss [47].

Machine learning methods are widely utilized in biomedical sciences. Convolutional neural networks (CNNs), a form of deep learning methods, are optimized for analyzing and interpreting visual information. CNNs are trained on datasets labeled by experts, enabling them to recognize visual patterns that are associated with anatomical structures in hierarchical order [48]. Models based on convolutional neural networks have broad applications in dental radiology, including teeth detection and the assessment of alveolar bone loss. Ozcelik et al. proposed a deep learning method for teeth segmentation named SE-IB-ED. The authors achieved an accuracy of 92.84% and a recall of 99.92%, demonstrating a high efficacy of teeth identification on panoramic radiographs [49]. Another model based on convolutional neural networks named Mask-RCNN achieved an accuracy of 98.4% in teeth classification [50]. Mohammad et al. utilized a deep learning CNN in teeth segmentation at various stages of development. The authors achieved an accuracy of 97.74% on training data and 78.13% on a testing set. The results generated by the model were in moderate agreement with human evaluators [51]. The highest sensitivity among the ChatGPT versions tested in our study was achieved by ChatGPT o4-mini-high, which reached 93.1%, followed by ChatGPT o1 with 89.7%, while ChatGPT 4.5 and ChatGPT o3 exhibited the same value of 85.8%. However, the models differed in the values of specificity. ChatGPT o1 most frequently identified missing teeth as present. In terms of predictive positive value, ChatGPT o3 reached the highest value of 94%. The models 4.5 and o3 achieved the highest values of positive predictive value (PPV) and specificity, indicating their reliability in minimizing the false positive identification of teeth. ChatGPT 4.5, o3 and o4-mini-high were in substantial agreement with the clinicians, while ChatGPT o1 presented moderate agreement with the examiners. Considering that the ChatGPT models have not been trained on annotated datasets and their performance relies on self-supervised learning, the results are considered promising. ChatGPT has the potential to serve in the future as a tool to support the automated evaluation of teeth presence in multidisciplinary treatment planning, as well as for statistical purposes. Clinical supervision is advised in the context of the current performance of the models. The increasing volume of training data is expected to enhance the clinical potential of ChatGPT. However, it is essential to emphasize that the validation of ChatGPT models for clinical applications requires acceptance from the medical community and the establishment in official guidelines.

The assessment of the RBH on the approximal surfaces performed by clinicians demonstrated excellent agreement. Each clinician’s measurements were closely aligned with others (ICC (2,1) = 0.96 for the distal surfaces; ICC (2,1) = 0.95 for the mesial surfaces), as well as the mean value of the three clinicians (ICC (2,3) = 0.99 for the distal surfaces; ICC (2,3) = 0.98 for the mesial surfaces). The high repeatability of the results was related to the precise protocol, clearly defined criteria and reference points. The consistency of the RBH measurements between the clinicians was further supported by the absence of statistically significant differences between the measurements on the distal and mesial surfaces (*p* = 0.7853 and *p* = 0.7933, respectively). RBH measurements conducted by clinicians provide a reliable reference for evaluating the performance of ChatGPT models. Nevertheless, there were statistically significant differences in the measurements of the RBH between the ChatGPT models (*p* < 0.0001 for the mesial and distal surfaces). Considering the standardization involving textual instructions, the differences in the RBH analysis were not related to the heterogeneity of the input data. The factors responsible for the discrepancies in RBH measurement may include the diversity of image processing paradigms and differences in the capacities of the built-in databases.

One of the key factors that influence ChatGPT performance is its internal architecture and data management strategy. Version 4.5 is focused on scaling unsupervised learning, while versions o1, o3 and o4-mini-high (also referred to as o-series) process data through a chain of thought. ChatGPT 4.5 is more efficient in general knowledge operations requiring a conversational, creative, human-like approach. The o-series is more efficient at reasoning and quantitative analysis, including STEM and logic queries [52]. Both version 4.5 and the o-series incorporate file uploads, including images. Despite a growing interest in the AI implementation in medical science, there is a notable lack of literature depicting visual data analysis with the use of ChatGPT. However, according to the OpenAI developers, models o3 and o4-mini (the older version of o4-mini-high) present the most promising accuracy in the perception of basic visual parameters, including edges, colors and brightness in comparison with models o1 and 4o (90.1%, 87.3%, 57%, 50.4%, respectively). Similarly, the models o3 and o4-mini presented the highest efficacy in tasks combining reasoning and a visual search in comparison with model o1 and 4o (95.7%, 94.6%, 69.7%, 73.9%, respectively) [53]. In our study, we observed an opposing tendency in the RBH measurement on panoramic radiographs. Among the o-series, model o1 achieved the lowest mean bias, followed by o3 and o4-mini-high (+13 pp, +16 pp and +20 pp for the distal surfaces, respectively; +11 pp, +15 pp and +19 pp for the mesial surfaces, respectively). Moreover, ChatGPT 4.5 demonstrated the greatest agreement with the clinicians’ results (bias: +12 pp for the distal surfaces; +11 pp for the mesial surfaces). ChatGPT 4.5 achieved the highest accuracy in the RBH assessment among the studied versions, although the o1 model generated only a slightly higher bias. According to Ryden et al., patients with a mean RBH > 80% are classified as healthy, while below the threshold are considered cases of periodontitis [10]. Machado et al. demonstrated in their study that the R-PBL, which is equivalent to the RBH, could be effectively applied to periodontitis screening [35]. Considering that all models tested in our study consistently overestimated the RBH in comparison with the clinicians’ measurements, ChatGPT utilization in periodontal screening appears to be inherently associated with an inaccuracy that may lead to the meaningful underdiagnosis of periodontitis.

CNNs have been the subject of numerous scientific studies evaluating their high effectiveness in detecting periodontitis. In the study conducted by Krois et al., convolutional neural networks were trained on 2001 image segments from panoramic radiographs to measure periodontal bone loss. The segments were also evaluated by six dentists. Researchers found that there were no statistically significant differences in the mean accuracy between human examiners and CNNs (*p* = 0.067). Moreover, the CNNs presented a higher specificity than the clinicians (0.81 vs. 0.63), while the sensitivity was lower (0.81 vs. 0.92) [54]. Liu et al. presented a novel insight into the performance of a CNN in detecting periodontitis in comparison with human examiners, distinguishing between general dental practitioners and periodontal experts. In their research, a CNN achieved results comparable with periodontal experts and significantly outperformed general practitioners (0.800 vs. 0.813 vs. 0.693, respectively). This study suggests that a CNN may serve as valuable support for general dentists in their daily practice [55]. Xue et al. proposed a deep learning model integrating Mask R-CNN, TransUNet and YOLOv8 to evaluate the teeth position, bone loss, periodontitis classification and other parameters on panoramic images. The authors achieved diagnostic accuracy in nearly 9 out of 10 cases. The high PCC values of 0.832 (*p* < 0.001) and ICC values of 0.806 (*p* < 0.001) showed excellent repeatability and consistency with clinical evaluations [56]. CNNs trained on labeled data may represent a more effective approach to AI implementation in periodontal diagnostics compared with transformer-based models. However, robust conclusions require comparative studies conducted on the same dataset.

The literature contains a limited number of reports on the use of language models for assessing the bone level on dental panoramic images, particularly concerning the latest generations of multimodal ChatGPT models. Dasanayaka et al. introduced a complex method of generating reports of panoramic radiographs. The proposed model consisted of a large language model (LLama 3 8B) combined with an image-captioning model (BLIP-2). The authors achieved an accuracy of 88% in the assessment of bone loss, while the experts assigned a subjective rating of 7.5 out of 10 [57]. Silva et al. emphasized the technical complexity of manual data labeling, particularly in large-scale datasets. The authors proposed a four-stage strategy for automated labeling and the classification of dental conditions on panoramic images. The authors assessed 13 conditions on panoramic radiographs, including endodontic treatment, excess dental restorations, impacted teeth and residual roots. In the first step, the datasets were divided into the following categories: panoramic radiographs without labeling (RPR); panoramic radiographs with annotated data (O^2^PR); and panoramic images with textual reports (TRPR). Subsequently, the panoramic images were pseudolabeled using an instance segmentation network trained on the O^2^PR dataset, and tooth crops were generated. A Vision Transformer (ViT) was employed for classification pretraining, while an autolabeler was used for label extraction. GPT-4 was applied for the textual data extraction, which was subsequently linked with the corresponding images. In the final step, binary classifiers were trained to identify specific dental conditions. The authors demonstrated that cropped images with more contextual information were more accurately recognized by the classifier. The proposed model achieved a diagnostic accuracy comparable with a student in the final year of dental school [58]. The ChatGPT models tested in our study integrate capabilities for the visual interpretation of panoramic radiographs and automated report generation. Regarding their multimodal architecture, the evaluation of panoramic images is performed within a single input field, eliminating the need for intermediary systems.

The 95% CI limits of agreement width for the ChatGPT versions was bounded between 60 pp and 66 pp (for the distal surfaces). For the mesial surfaces the LoA width was narrower and ranged between 54 pp and 60 pp. Regarding the substantial LoA width for all the models, there was a risk that a single RBH measurement may lead to a misdiagnosis and an inadequate treatment plan. In periodontology, an inaccurate measurement of the RBH may lead to a wrong assignment of the stage and grade of periodontitis. Consequently, the therapeutic approach might lack essential elements that require precise indications, for example, regenerative surgery or resective periodontal surgery. In dental implantology, an appropriate periodontal examination is crucial in the treatment plan, particularly for patients with periodontitis. Periodontal disease could lead to the development of complications, including peri-implantitis [59]. Therefore, less accurate ChatGPT models should not be routinely applied in radiological diagnostics.

The Bland–Altman plot shows the presence of proportional bias characterized by larger positive discrepancies on the *Y*-axis for the lower mean RBH on the *X*-axis. In clinical context, this tendency could result in higher levels of bias in patients with more advanced periodontitis (lower RBH value) compared with individuals less affected by the disease (higher RBH value). To achieve an alignment with clinical judgment, a calibration procedure may be considered. Linear calibration could be applied by the subtraction of systematic bias from observed outcomes. For example, if the mean bias for ChatGPT 4.5 was 12 pp at the distal surfaces, this value would be subtracted to adjust the measurements. This method may be utilized in general statistical analyses, where the precise classification of healthy individuals and those affected by periodontitis is not necessary. However, for individual RBH measurements, a different statistical approach is required. The logarithmic transformation of RBH values prior to the main analysis and application of regression models enable the improvement of individual results in terms of the proportional bias.

The 2017 Classification of Periodontal and Peri-Implant Diseases and Conditions distinguishes between indirect and direct evidence to determine the periodontitis progression. The direct method refers to the increase in radiographic bone loss or clinical attachment loss over a 5-year period, measured in millimeters. Panoramic images cannot be utilized for this purpose due to the distortion and enlargement of anatomical structures, resulting in the inability to record accurate absolute measurements. In the indirect method, radiographic bone loss is estimated as a percentage and divided by the patient’s age [8]. The result is then compared with a reference scale. A single panoramic image provides an overview of both the upper and lower dental arches and allows for an indirect assessment of disease progression. In cases of diagnostic uncertainty, targeted periapical or bitewing radiographs may be performed. The Bland–Altman analysis demonstrated a positive mean bias and wide limits of agreement in the RBHs evaluated by the dental professionals and ChatGPT models. Since the grading system operated on narrow thresholds, ChatGPT-based assessments may lead to incorrect classification, particularly in estimating the rate of periodontitis progression.

An important aspect in the discussion of ChatGPT’s performance is the risk of “hallucinations” as a confounding factor. The phenomenon of hallucination describes a situation in which an AI model generates false data that appears to be credible [60]. The issue has been the subject of extensive research. However, the literature has predominantly focused on misinformation related to textual data processing [61,62,63]. In the context of the visual interpretation of radiographic images, there is a lack of scientific articles addressing the phenomenon. Hallucinations may be influenced by various factors, including the size and quality of the dataset used to train LLMs. This may arise from the fact that LLMs use a sequential prediction mechanism, which leads to the propagation of errors introduced during training. Moreover, LLMs are statistical models trained through unsupervised learning and are unable to determine the accuracy of the data [64]. ChatGPT is extensively shaped by Reinforcement Learning from Human Feedback (RLHF). This involves strengthening the responses that were rated by users as correct. According to Tlili and Burgos, hallucinations may also originate from human cognitive biases and stereotypical thinking [65]. Therefore, RLHF could also contribute to the reproduction of errors. The risk of hallucinations may be mitigated through the implementation of a well-constructed prompt [66]. In our study, we applied a comprehensive prompt that precisely defined the required information, as well as the expected structure of the output.

The utilization of LLMs in new areas should be considered in the context of environmental costs. Shaji George et al. indicated that the training of ChatGPT-3 may have been associated with a substantial consumption of water [67]. Given that the latest ChatGPT models operate on significantly larger datasets, the environmental impact of LLMs development is expected to be proportionally greater. Khowaja et al. highlighted the contribution of LLMs to the carbon footprint associated with energy consumption. Researchers estimated that the levels of carbon dioxide emissions may rise substantially as the number of newly developed LLMs increases, along with the expansion of training datasets and their growing popularity [68]. Yoon et al. compared the energy consumption patterns of convolutional neural networks (CNNs) and large language models (LLMs). The authors found that with increasing batch size, CNNs consumed proportionally more energy for convolutional computations than for memory access. LLMs, in contrast, primarily consumed energy on memory-related operations [69]. An increase in the number of parameters processed by ChatGPT is likely to result in higher memory-related energy consumption. Various strategies have been proposed to challenge excessive energy consumption associated with the development of transformer models. Irugalbandara et al. suggested employing small language models (SLMs) for processing qualitative and quantitative data. In comparison with ChatGPT-4, SLMs presented a comparable performance, accompanied by a substantial reduction in costs [70]. Local implementation of LLMs, for instance, in healthcare facilities, represents one of the cost-effective solutions that do not require the maintenance of an expensive computational infrastructure [71]. Stojkovic et al. proposed an energy management model for LLMs, referred to as DynamoLLM. The authors achieved a 52% reduction in energy consumption and a 61% reduction in operational costs [72]. In our study, we employed the most advanced ChatGPT models to perform complex tasks involving text and image processing. The economic and environmental costs associated with multimodal queries should be considered in the rational implementation of AI in healthcare.

Multimodal language models are characterized by broad accessibility and ease of use, largely due to their ability to process natural, conversational language. These models can generate complex, structured and seemingly reliable responses. As a result, patients may be encouraged to use ChatGPT for self-diagnosis. However, this approach poses a significant risk of misinterpretation and improper health-related decisions. Similarly, healthcare professionals seeking to compensate for knowledge gaps or to accelerate the diagnostic process may also be at risk, particularly in the absence of the critical evaluation of the model’s outputs. In our study, we demonstrated that ChatGPT, as a general-purpose tool operating solely on its internal knowledge base, cannot be routinely applied for the diagnosis of periodontitis. The continuous development of internal training datasets and targeted training in specific clinical contexts and the implementation of Reinforcement Learning with Human Feedback (RLHF) have the potential to improve the clinical utility of ChatGPT models. However, further investigation is advised in this field.

The development of this publication involved several limitations:

1. Panoramic images were used in their original form, even though numerous publications propose various preprocessing methods. Available techniques such as contrast enhancement, filter application, image cropping or image sharpening were not employed.
2. We analyzed a limited number of panoramic radiographs due to the variability in image quality and strict exclusion criteria. However, this approach ensured that the results were not influenced by confounding factors and accurately reflected the actual effectiveness of ChatGPT.
3. Three dentists participated in the research, while other studies involved a greater number of clinicians, including radiologists.
4. There is a lack of comparable data in the existing scientific literature assessing the effectiveness of medical image analysis performed by ChatGPT. Additionally, the OpenAI developers have not provided any reports on the clinical application of ChatGPT models.
5. A highly complex prompt may challenge the models’ performance in terms of processing a large set of logically connected words. However, the implementation of precise instructions reduces the risk of hallucinations.
6. The risk of hallucinations related to the built-in training database.

## 5. Conclusions

1. In the assessment of tooth counts, the models mainly showed substantial agreement with the clinicians. ChatGPT o4-mini-high showed the highest sensitivity. However, based on the overall evaluation of all the parameters, models 4.5 and o3 proved to be the most reliable. In comparison with various convolutional neural networks, the ChatGPT models achieved similar or lower performances. Currently, transformer models are not recommended for the independent analysis of teeth presence.
2. The clinicians’ measurements of the RBH presented excellent consistency, which provided a reliable reference for comparison with the ChatGPT performance.
3. The ChatGPT models exhibited significant differences in the assessment of the RBH on the approximal surfaces.
4. ChatGPT o1 demonstrated the lowest mean bias in the RBH measurement among the o-series. The lowest mean bias was observed in ChatGPT 4.5 compared with all the other models. The RBH values yielded by all the models fell within wide limits of agreement. This may be clinically relevant in the context of single measurements that could significantly deviate from clinician evaluations.
5. A proportional bias was observed across all the ChatGPT models with a higher mean bias associated with a lower mean RBH. This may have clinical implications for the underdiagnosis of periodontal disease.
6. ChatGPT 4.5 achieved the highest reliability in both the assessment of tooth counts and the RBH measurement between the ChatGPT models, suggesting its greatest potential for clinical utilization. However, ChatGPT 4.5 remains a preview version of the most advanced generative pre-trained transformer and may undergo substantial reconstruction.
7. Convolutional neural networks currently appear to outperform ChatGPT models in the assessment of the alveolar bone level and associated bone loss. However, further studies are needed to compare the effectiveness of both AI models.
8. As multimodal language models continue to develop and different calibration methods may be applied, ChatGPT’s potential in assessing panoramic images is increasing. Currently, ChatGPT models demonstrate inadequate agreement with dental professionals in assessing the RBH to serve as a diagnostic tool. Models 4.5 and o3 show potential in tooth count evaluation but are not precise enough for clinical implementation.
9. Hardware and environmental costs should be a significant component of the responsible implementation of transformer models in dental diagnostics.
10. Multimodal language models are accessible and widely available for various applications. However, in highly specialized fields, such as periodontology, they may pose a risk to individuals who are unable to verify the reliability of the outputs.

## Figures and Tables

**Table 1:** Number of teeth based on panoramic image assessment.

Clinician 1–3	ChatGPT 4.5	ChatGPT o1	ChatGPT o3	ChatGPT o4-Mini-High
**232 (72.5%)**	214 (66.9%)	243 (75.9%)	212 (66.3%)	239 (74.7%)
				
	Clinician vs. ChatGPT 4.5	Clinician vs. ChatGPT o1	Clinician vs. ChatGPT o3	Clinician vs. ChatGPT o4-mini-high
**Fleiss’ kappa (95% CI)**	0.65 (0.56–0.73)	0.52 (0.41–0.62)	0.66 (0.57–0.75)	0.69 (0.59–0.78)
**Sensitivity**	85.8%	89.7%	85.8%	93.1%
**Specificity**	83.0%	60.2%	85.2%	73.9%
**PPV**	93.0%	85.6%	94.0%	90.4%

PPV—predictive positive value; CI—confidence interval.

**Table 2:** Intraclass correlation coefficient for RBH values at approximal surfaces.

Approximal Surface	Number of Measurements	ICC (2,1) (95% CI)	ICC (2,3) (95% CI)
**Distal**	142	0.96 (0.94–0.97)	0.99 (0.98–0.99)
**Mesial**	147	0.95 (0.93–0.96)	0.98 (0.98–0.99)

ICC—intraclass correlation coefficient; CI—confidence interval.

**Table 3:** RBH values for distal surfaces—clinicians’ evaluations.

	Clinician 1	Clinician 2	Clinician 3	*p*-Value
				0.7853
**Mean (SD)**	70.4% (11.6%)	72.6% (12.4%)	71.9% (12.8%)	
**Range**	28.0–85.0%	31.0–86.0%	26.0–89.0%	
**Median (IQR)**	72.0% (16.0%)	75.0% (18.5%)	75.5% (15.0%)	
**95% CI**	[66.2%; 74.6%]	[68.2%; 77.1%]	[67.3%; 76.5%]	

SD—standard deviation; IQR—interquartile range; CI—confidence interval.

**Table 4:** RBH values for mesial surfaces—clinicians’ evaluation.

	Clinician 1	Clinician 2	Clinician 3	*p*-Value
				0.7933
**Mean (SD)**	68.8% (9.1%)	70.7% (9.5%)	69.7% (9.2%)	
**Range**	52.0–85.0%	56.0–87.0%	54.0–90.0%	
**Median (IQR)**	69.0% (14.0%)	73.0% (16.0%)	71.0% (12.0%)	
**95% CI**	[65.5%; 72.1%]	[67.2%; 74.2%]	[66.4%; 73.1%]	

SD—standard deviation; IQR—interquartile range; CI—confidence interval.

**Table 5:** RBH values for distal surfaces—ChatGPT models’ evaluation.

	ChatGPT 4.5	ChatGPT o1	ChatGPT o3	ChatGPT o4-Mini-High	*p*-Value
					<0.0001
**Mean (SD)**	73.7% (6.9%) ^a,b^	76.7% (15.4%) ^c,d^	73.0% (12.4%) ^a,c^	77.0% (10.4%) ^b,d^	^a,b,d^ <0.0001 ^c^ 0.0053
**Range**	60.0–85.0%	40.0–90.0%	60.0–92.0%	50.0–90.0%	
**Median (IQR)**	74.0% (10.0%)	85.0% (20.0%)	70.0% (25.0%)	80.0% (5.0%)	
**95% CI**	[70.9%; 76.6%]	[69.6%; 83.7%]	[67.6%; 78.4%]	[72.9%; 81.2%]	

SD—standard deviation; IQR—interquartile range; CI—confidence interval; ^a–d^ post-hoc test.

**Table 6:** RBH values for mesial surfaces—ChatGPT models’ evaluation.

	ChatGPT 4.5	ChatGPT o1	ChatGPT o3	ChatGPT o4-Mini-High	*p*-Value
					<0.0001
**Mean (SD)**	74.2% (6.8%) ^a,b^	75.8% (16.3%) ^c,d^	74.2% (12.4%) ^a,c^	78.2% (10.5%) ^b,d^	^a,b,d^ <0.0001 ^c^ 0.0002
**Range**	55.0–85.0%	35.0–90.0%	60.0–92.0%	45.0–92.0%	
**Median (IQR)**	75.0% (10.0%)	85.0% (25.0%)	70.0% (25.0%)	80.0% (0.0%)	
**95% CI**	[71.4%; 77.1%]	[68.1%; 83.4%]	[68.9%; 79.6%]	[74.0%; 82.5%]	

SD—standard deviation; IQR—interquartile range; CI—confidence interval; ^a–d^ post-hoc test.

**Table 7:** Mean biases and limits of agreement for Bland–Altman analysis between ChatGPT models and clinicians for RBH measurement for distal surfaces.

Clinician	ChatGPT 4.5 Bias (95% CI)	ChatGPT o1 Bias (95% CI)	ChatGPT o3 Bias (95% CI)	ChatGPT o4-Mini-High Bias (95% CI)
**1**	+13 pp (−18 pp; +43 pp)	+13 pp (−20 pp; +46 pp)	+17 pp (−16 pp; +50 pp)	+21 pp (−11 pp; +52 pp)
**2**	+12 pp (−19 pp; +43 pp)	+13 pp (−22 pp; +47 pp)	+16 pp (−18 pp; +50 pp)	+20 pp (−13 pp; +53 pp)
**3**	+12 pp (−18 pp; +42 pp)	+12 pp (−22 pp; +46 pp)	+16 pp (−17 pp; +49 pp)	+20 pp (−12 pp; +51 pp)

pp—percentage points; CI—confidence interval.

**Table 8:** Mean bias and limits of agreement for Bland–Altman analysis between ChatGPT models and clinicians for RBH measurement for mesial surfaces.

Clinician	ChatGPT 4.5 Bias (95% CI)	ChatGPT o1 Bias (95% CI)	ChatGPT o3 Bias (95% CI)	ChatGPT o4-Mini-High Bias (95% CI)
**1**	+12 pp (−15 pp; +38 pp)	+11 pp (−19 pp; +41 pp)	+16 pp (−13 pp; +44 pp)	+19 pp (−8 pp; +46 pp)
**2**	+11 pp (−17 pp; +38 pp)	+11 pp (−19 pp; +40 pp)	+15 pp (−14 pp; +44 pp)	+18 pp (−8 pp; +45 pp)
**3**	+11 pp (−15 pp; +38 pp)	+11 pp (−19 pp; +42 pp)	+16 pp (−13 pp; +45 pp)	+19 pp (−7 pp; +45 pp)

pp—percentage points; CI—confidence interval.

**Table 9:** Comparison of ChatGPT models’ performance in RBH measurement for distal surfaces.

Model	Bias	LoA Width	Performance
**4.5**	+12 pp	~60 pp	Lowest bias
**o1**	+13 pp	~66 pp	Moderate bias
**o3**	+16 pp	~66 pp	Moderate bias
**o4-mini-high**	+20 pp	~64 pp	Highest bias

pp—percentage points; LoA—limit of agreement.

**Table 10:** Comparison of ChatGPT models’ performance in RBH measurement for mesial surfaces.

Model	Bias	LoA Width	Performance
**4.5**	+11 pp	~54 pp	Lowest bias
**o1**	+11 pp	~60 pp	Wider LoA
**o3**	+15 pp	~58 pp	Moderate bias
**o4-mini-high**	+19 pp	~54 pp	Highest bias

pp—percentage points; LoA—limit of agreement.

## Data Availability

The data presented in this study are available in the Supplementary Materials ChatGPT_Reports.zip.

## Supplementary Materials

The following supporting information can be downloaded from https://www.mdpi.com/article/10.3390/diagnostics15151851/s1. The Supplementary Materials include reports generated by the ChatGPT models ChatGPT_Reports.zip.

## Abbreviations

The following abbreviations are used in this manuscript:

RBH	Remaining bone height
CEJ	Cemento-enamel junction
LLMs	Large language models
ChatGPT	Chat Generative Pre-Trained Transformer
CNNs	Convolutional neural networks
CBCT	Cone-beam computed tomography
LoAs	Limits of agreement
PPV	Predictive positive value
ICC	Intraclass correlation coefficient
Pp	Percentage points
RLHF	Reinforcement Learning from Human Feedback
SLMs	Small language models
RPR	Raw panoramic radiograph
O^2^PR	O^2^ panoramic radiograph
TRPR	Textual report with panoramic radiographs
ViT	Visual Transformer
GPT-4	Generative Pre-Trained Transformer-4
