# Supplementary material for: Advances in Periodontal Diagnostics: Application of MultiModal Language Models in Visual Interpretation of Panoramic Radiographs
Source: Diagnostics (Basel). 2025 Jul 23;15(15):1851. doi: 10.3390/diagnostics15151851 (PMC12345756; doi:10.3390/diagnostics15151851)
Supplement: Supplementary file 1 [file diagnostics-15-01851-s001.zip › ChatGPT_Reports.zip!/4.5.zip!/10_ChatGPT_4.5.pdf]

Radiological Report

Table no. 1 - Evaluation of the presence of teeth:

Tooth no.	Tooth Presence
18	0
17	1
16	1
15	1
14	1
13	1
12	1
11	1
21	1
22	1
23	1
24	1
25	1
26	1
27	1
28	0
38	0
37	1
36	1
35	1
34	1
33	1
32	1
31	1
41	1
42	1
43	1
44	1
45	1
46	1
47	1
48	0

Table no. 2 - Evaluation of the remaining bone height (RBH):

Approximal surfaces	Remaining bone height
18D	X
18M	X
17D	75%
17M	80%
16D	82%
16M	85%
15D	86%
15M	87%
14D	85%
14M	83%
13D	88%
13M	90%
12D	89%
12M	90%
11D	91%
11M	92%
21M	91%
21D	90%
22M	89%
22D	88%
23M	87%
23D	86%
24M	85%
24D	84%
25M	85%
25D	86%
26M	87%
26D	88%
27M	82%
27D	80%
28M	X
28D	X
38D	X
38M	X
37D	78%
37M	80%

36D	83%
36M	85%
35D	86%
35M	87%
34D	85%
34M	86%
33D	88%
33M	89%
32D	90%
32M	91%
31D	92%
31M	93%
41M	92%
41D	91%
42M	90%
42D	89%
43M	88%
43D	87%
44M	85%
44D	86%
45M	87%
45D	88%
46M	84%
46D	83%
47M	82%
47D	80%
48M	X
48D	X
