# Supplementary material for: Advances in Periodontal Diagnostics: Application of MultiModal Language Models in Visual Interpretation of Panoramic Radiographs
Source: Diagnostics (Basel). 2025 Jul 23;15(15):1851. doi: 10.3390/diagnostics15151851 (PMC12345756; doi:10.3390/diagnostics15151851)
Supplement: Supplementary file 1 [file diagnostics-15-01851-s001.zip › ChatGPT_Reports.zip!/4.5.zip!/1_ChatGPT_4.5.pdf]

Radiological Report

Table no. 1 - Evaluation of the Presence of Teeth:

Tooth no.	Tooth Presence
18	0
17	1
16	1
15	1
14	1
13	1
12	1
11	1
21	1
22	1
23	1
24	1
25	1
26	1
27	1
28	0
38	0
37	1
36	1
35	1
34	1
33	1
32	1
31	1
41	1
42	1
43	1
44	1
45	1
46	1
47	1
48	0

Table no. 2 - Evaluation of Remaining Bone Height (RBH):

Approximal surfaces	Remaining bone height
18D	X
18M	X
17D	85%
17M	80%
16D	80%
16M	78%
15D	85%
15M	83%
14D	80%
14M	78%
13D	85%
13M	88%
12D	90%
12M	88%
11D	90%
11M	89%
21M	89%
21D	88%
22M	90%
22D	87%
23M	85%
23D	83%
24M	80%
24D	78%
25M	80%
25D	79%
26M	78%
26D	77%
27M	82%
27D	X
28M	X
28D	X
38D	X
38M	X
37D	82%
37M	80%

36D	79%
36M	78%
35D	85%
35M	83%
34D	88%
34M	87%
33D	89%
33M	88%
32D	90%
32M	88%
31D	90%
31M	89%
41M	89%
41D	88%
42M	90%
42D	88%
43M	87%
43D	86%
44M	85%
44D	84%
45M	83%
45D	82%
46M	80%
46D	78%
47M	77%
47D	X
48M	X
48D	X
