# Supplementary material for: Advances in Periodontal Diagnostics: Application of MultiModal Language Models in Visual Interpretation of Panoramic Radiographs
Source: Diagnostics (Basel). 2025 Jul 23;15(15):1851. doi: 10.3390/diagnostics15151851 (PMC12345756; doi:10.3390/diagnostics15151851)
Supplement: Supplementary file 1 [file diagnostics-15-01851-s001.zip › ChatGPT_Reports.zip!/4.5.zip!/2_ChatGPT_4.5.pdf]

Radiological Report

Table no. 1 - Evaluation of the presence of teeth

Tooth no.	Tooth Presence
18	0
17	1
16	1
15	1
14	1
13	1
12	1
11	1
21	1
22	1
23	1
24	1
25	0
26	1
27	1
28	0
38	0
37	0
36	0
35	0
34	0

33	1
32	1
31	1
41	1
42	1
43	1
44	1
45	0
46	0
47	0
48	0

Table no. 2 - Evaluation of the remaining bone height (RBH)

Approximal surfaces	Remaining bone height
18D	X
18M	X
17D	65%
17M	70%
16D	60%
16M	65%
15D	75%
15M	80%
14D	70%
14M	75%
13D	85%
13M	80%

12D	80%
12M	85%
11D	85%
11M	80%
21M	80%
21D	85%
22M	85%
22D	80%
23M	80%
23D	85%
24M	80%
24D	85%
25M	X
25D	X
26M	75%
26D	80%
27M	70%
27D	75%
28M	X
28D	X
38D	X
38M	X
37D	X
37M	X
36D	X
36M	X

35D	X
35M	X
34D	X
34M	X
33D	70%
33M	75%
32D	75%
32M	70%
31D	70%
31M	75%
41M	75%
41D	70%
42M	80%
42D	75%
43M	85%
43D	80%
44M	75%
44D	70%
45M	X
45D	X
46M	X
46D	X
47M	X
47D	X
48M	X
48D	X
