# Supplementary material for: Advances in Periodontal Diagnostics: Application of MultiModal Language Models in Visual Interpretation of Panoramic Radiographs
Source: Diagnostics (Basel). 2025 Jul 23;15(15):1851. doi: 10.3390/diagnostics15151851 (PMC12345756; doi:10.3390/diagnostics15151851)
Supplement: Supplementary file 1 [file diagnostics-15-01851-s001.zip › ChatGPT_Reports.zip!/4.5.zip!/3_ChatGPT_4.5.pdf]

Tooth no.	Tooth Presence
18	0
17	1
16	1
15	1
14	1
13	1
12	1
11	1
21	1
22	1
23	1
24	1
25	1
26	1
27	1
28	0
38	0
37	1
36	1
35	0
34	0
33	1
32	1
31	1
41	1
42	1
43	1
44	0
45	0
46	1
47	1
48	0

Approximal surfaces	Remaining bone height
18D	N/A
18M	N/A
17D	60%
17M	65%
16D	65%
16M	65%
15D	70%
15M	70%
14D	70%
14M	70%
13D	80%
13M	80%
12D	80%
12M	80%
11D	85%
11M	85%
21M	85%
21D	85%
22M	80%
22D	80%
23M	80%
23D	80%
24M	75%
24D	75%
25M	75%
25D	75%
26M	65%
26D	65%
27M	60%
27D	60%
28M	N/A
28D	N/A
38D	N/A
38M	N/A
37D	65%
37M	65%
36D	70%

Approximal surfaces	Remaining bone height
36M	70%
35D	N/A
35M	N/A
34D	N/A
34M	N/A
33D	80%
33M	80%
32D	80%
32M	80%
31D	80%
31M	80%
41M	80%
41D	80%
42M	80%
42D	80%
43M	80%
43D	80%
44M	N/A
44D	N/A
45M	N/A
45D	N/A
46M	70%
46D	70%
47M	65%
47D	65%
48M	N/A
48D	N/A
