# Supplementary material for: Advances in Periodontal Diagnostics: Application of MultiModal Language Models in Visual Interpretation of Panoramic Radiographs
Source: Diagnostics (Basel). 2025 Jul 23;15(15):1851. doi: 10.3390/diagnostics15151851 (PMC12345756; doi:10.3390/diagnostics15151851)
Supplement: Supplementary file 1 [file diagnostics-15-01851-s001.zip › ChatGPT_Reports.zip!/4.5.zip!/5_ChatGPT_4.5.pdf]

Radiological Report

Table no. 1 - Evaluation of the presence of teeth

Tooth no.	Tooth Presence
18	0
17	1
16	0
15	0
14	0
13	0
12	0
11	0
21	0
22	0
23	0
24	0
25	0
26	0
27	0
28	0
38	0
37	0
36	1
35	0
34	0
33	0
32	1
31	1
41	1
42	1
43	1
44	1
45	0
46	0
47	0
48	0

Table no. 2 - Evaluation of the remaining bone height (RBH)

Approximal surfaces	Remaining bone height
18D	X
18M	X
17D	60%
17M	55%
16D	X

16M	X
15D	X
15M	X
14D	X
14M	X
13D	X
13M	X
12D	X
12M	X
11D	X
11M	X
21M	X
21D	X
22M	X
22D	X
23M	X
23D	X
24M	X
24D	X
25M	X
25D	X
26M	X
26D	X
27M	X
27D	X
28M	X
28D	X
38D	X
38M	X
37D	X
37M	X
36D	70%
36M	65%
35D	X
35M	X
34D	X
34M	X
33D	X
33M	X
32D	85%
32M	80%
31D	80%
31M	75%
41M	75%
41D	70%
42M	70%
42D	70%
43M	75%

43D	75%
44M	70%
44D	70%
45M	X
45D	X
46M	X
46D	X
47M	X
47D	X
48M	X
48D	X
