# Supplementary material for: Advances in Periodontal Diagnostics: Application of MultiModal Language Models in Visual Interpretation of Panoramic Radiographs
Source: Diagnostics (Basel). 2025 Jul 23;15(15):1851. doi: 10.3390/diagnostics15151851 (PMC12345756; doi:10.3390/diagnostics15151851)
Supplement: Supplementary file 1 [file diagnostics-15-01851-s001.zip › ChatGPT_Reports.zip!/4.5.zip!/6_ChatGPT_4.5.pdf]

Radiological Report

Table no. 1 - Evaluation of the presence of teeth

Tooth no.	Tooth Presence
18	0
17	1
16	1
15	1
14	1
13	1
12	1
11	1
21	1
22	1
23	1
24	1
25	1
26	1
27	1
28	0
38	0
37	1
36	1
35	1
34	1
33	1

32	1
31	1
41	1
42	1
43	1
44	1
45	1
46	1
47	1
48	0

Table no. 2 - Evaluation of the remaining bone height (RBH)

Approximal surfaces	Remaining bone height (%)
18D	X
18M	X
17D	88
17M	90
16D	90
16M	92
15D	93
15M	92
14D	92
14M	91
13D	91
13M	92
12D	92

12M	93
11D	94
11M	94
21M	94
21D	93
22M	93
22D	92
23M	91
23D	91
24M	91
24D	90
25M	90
25D	91
26M	91
26D	90
27M	89
27D	88
28M	X
28D	X
38D	X
38M	X
37D	89
37M	90
36D	91
36M	90
35D	92

35M	91
34D	91
34M	92
33D	92
33M	93
32D	93
32M	94
31D	93
31M	92
41M	92
41D	92
42M	91
42D	91
43M	91
43D	90
44M	90
44D	89
45M	89
45D	90
46M	90
46D	89
47M	88
47D	87
48M	X
48D	X
