# Supplementary material for: Advances in Periodontal Diagnostics: Application of MultiModal Language Models in Visual Interpretation of Panoramic Radiographs
Source: Diagnostics (Basel). 2025 Jul 23;15(15):1851. doi: 10.3390/diagnostics15151851 (PMC12345756; doi:10.3390/diagnostics15151851)
Supplement: Supplementary file 1 [file diagnostics-15-01851-s001.zip › ChatGPT_Reports.zip!/4.5.zip!/7_ChatGPT_4.5.pdf]

Table no. 1 - Evaluation of the presence of teeth

Tooth no.	Tooth Presence
18	0
17	1
16	1
15	1
14	1
13	1
12	1
11	1
21	1
22	1
23	1
24	1
25	1
26	1
27	1
28	0
38	0
37	1
36	1
35	1
34	1
33	1
32	1

31	1
41	1
42	1
43	1
44	1
45	1
46	1
47	1
48	0

Table no. 2 - Evaluation of the remaining bone height (RBH)

Approximal surfaces	Remaining bone height
18D	X
18M	X
17D	80%
17M	85%
16D	78%
16M	82%
15D	80%
15M	83%
14D	82%
14M	80%
13D	84%
13M	85%

12D	83%
12M	82%
11D	85%
11M	86%
21M	86%
21D	85%
22M	83%
22D	84%
23M	82%
23D	81%
24M	80%
24D	79%
25M	80%
25D	78%
26M	77%
26D	76%
27M	75%
27D	X
28M	X
28D	X
38D	X
38M	X
37D	79%
37M	81%
36D	80%
36M	82%

35D	78%
35M	80%
34D	82%
34M	83%
33D	81%
33M	80%
32D	78%
32M	77%
31D	76%
31M	77%
41M	79%
41D	80%
42M	82%
42D	81%
43M	80%
43D	79%
44M	78%
44D	77%
45M	75%
45D	76%
46M	78%
46D	79%
47M	X
47D	X
48M	X
48D	X
