# Supplementary material for: Advances in Periodontal Diagnostics: Application of MultiModal Language Models in Visual Interpretation of Panoramic Radiographs
Source: Diagnostics (Basel). 2025 Jul 23;15(15):1851. doi: 10.3390/diagnostics15151851 (PMC12345756; doi:10.3390/diagnostics15151851)
Supplement: Supplementary file 1 [file diagnostics-15-01851-s001.zip › ChatGPT_Reports.zip!/4.5.zip!/8_ChatGPT_4.5.pdf]

Radiological Report

Table no. 1 - Evaluation of the presence of teeth

Tooth no.	Tooth Presence
18	0
17	1
16	1
15	1
14	1
13	1
12	1
11	1
21	1
22	1
23	1
24	1
25	1
26	1
27	1
28	0
38	0
37	1
36	1
35	1
34	1
33	1

32	1
31	1
41	1
42	1
43	1
44	1
45	1
46	1
47	1
48	0

Table no. 2 - Evaluation of the remaining bone height (RBH)

Approximal surfaces	Remaining bone height
18D	X
18M	X
17D	90
17M	92
16D	85
16M	87
15D	85
15M	86
14D	88
14M	90
13D	92
13M	93
12D	92

12M	94
11D	90
11M	91
21M	91
21D	90
22M	93
22D	94
23M	92
23D	91
24M	89
24D	88
25M	87
25D	85
26M	90
26D	89
27M	88
27D	87
28M	X
28D	X
38D	X
38M	X
37D	88
37M	87
36D	89
36M	90
35D	92

35M	91
34D	90
34M	89
33D	93
33M	92
32D	94
32M	93
31D	92
31M	91
41M	90
41D	91
42M	89
42D	90
43M	91
43D	92
44M	88
44D	89
45M	90
45D	88
46M	87
46D	85
47M	86
47D	85
48M	X
48D	X
