# Supplementary material for: Advances in Periodontal Diagnostics: Application of MultiModal Language Models in Visual Interpretation of Panoramic Radiographs
Source: Diagnostics (Basel). 2025 Jul 23;15(15):1851. doi: 10.3390/diagnostics15151851 (PMC12345756; doi:10.3390/diagnostics15151851)
Supplement: Supplementary file 1 [file diagnostics-15-01851-s001.zip › ChatGPT_Reports.zip!/4.5.zip!/9_ChatGPT_4.5.pdf]

Radiological Report

Table no. 1 - Evaluation of the presence of teeth

Tooth no.	Tooth Presence
18	0
17	0
16	0
15	0
14	0
13	1
12	1
11	1
21	1
22	1
23	1
24	0
25	0
26	0
27	0
28	0
38	0
37	0
36	0
35	0
34	0

33	1
32	1
31	1
41	1
42	1
43	1
44	0
45	0
46	0
47	0
48	0

Table no. 2 - Evaluation of the remaining bone height (RBH)

Approximal surfaces	Remaining bone height (%)
18D	X
18M	X
17D	X
17M	X
16D	X
16M	X
15D	X
15M	X
14D	X
14M	X

13D	75
13M	80
12D	70
12M	72
11D	68
11M	70
21M	70
21D	68
22M	72
22D	70
23M	78
23D	76
24M	X
24D	X
25M	X
25D	X
26M	X
26D	X
27M	X
27D	X
28M	X
28D	X
38D	X
38M	X
37D	X
37M	X

36D	X
36M	X
35D	X
35M	X
34D	X
34M	X
33D	74
33M	72
32D	76
32M	78
31D	80
31M	82
41M	80
41D	82
42M	78
42D	76
43M	72
43D	74
44M	X
44D	X
45M	X
45D	X
46M	X
46D	X
47M	X
47D	X

48M	X
48D	X
