# Supplementary material for: Advances in Periodontal Diagnostics: Application of MultiModal Language Models in Visual Interpretation of Panoramic Radiographs
Source: Diagnostics (Basel). 2025 Jul 23;15(15):1851. doi: 10.3390/diagnostics15151851 (PMC12345756; doi:10.3390/diagnostics15151851)
Supplement: Supplementary file 1 [file diagnostics-15-01851-s001.zip › ChatGPT_Reports.zip!/o1.zip!/1_ChatGPT_o1.pdf]

2. Table No. 1 – Evaluation of the Presence of Teeth

Tooth no.	Tooth Presence
18	0
17	1
16	1
15	1
14	1
13	1
12	1
11	1
21	1
22	1
23	1
24	1
25	1
26	1
27	1
28	0
38	1
37	1
36	1
35	1
34	1
33	1
32	1
31	1
41	1
42	1
43	1
44	1
45	1
46	1
47	1
48	1

**Approximal surfaces**	**Remaining bone height**
18D	X
18M	X
17D	85%
17M	85%
16D	85%
16M	85%
15D	85%
15M	85%
14D	85%
14M	85%
13D	90%
13M	90%
12D	90%
12M	90%
11D	90%
11M	90%
21M	90%
21D	90%
22M	90%
22D	90%
23M	90%
23D	90%
24M	85%
24D	85%
25M	85%
25D	85%
26M	85%
26D	85%
27M	85%
27D	85%
28M	X
28D	X
38D	75%
38M	75%
37D	85%
37M	85%
36D	80%

**Approximal surfaces**	**Remaining bone height**
36M	80%
35D	85%
35M	85%
34D	85%
34M	85%
33D	90%
33M	90%
32D	90%
32M	90%
31D	90%
31M	90%
41M	90%
41D	90%
42M	90%
42D	90%
43M	90%
43D	90%
44M	85%
44D	85%
45M	85%
45D	85%
46M	80%
46D	80%
47M	85%
47D	85%
48M	75%
48D	75%
