# Supplementary material for: Advances in Periodontal Diagnostics: Application of MultiModal Language Models in Visual Interpretation of Panoramic Radiographs
Source: Diagnostics (Basel). 2025 Jul 23;15(15):1851. doi: 10.3390/diagnostics15151851 (PMC12345756; doi:10.3390/diagnostics15151851)
Supplement: Supplementary file 1 [file diagnostics-15-01851-s001.zip › ChatGPT_Reports.zip!/o1.zip!/2_ChatGPT_o1.pdf]

Radiological Report

Table no. 1 - Evaluation of the presence of teeth

Tooth no.	Tooth Presence
18	0
17	0
16	0
15	0
14	1
13	1
12	1
11	1
21	1
22	1
23	1
24	1
25	0
26	0
27	0
28	0
38	0
37	0
36	0
35	1
34	1
33	1
32	1
31	1
41	1
42	1
43	1
44	1
45	1
46	1
47	0
48	0

Table no. 2 - Evaluation of the remaining bone height (RBH)

Approximal surfaces	Remaining bone height
18D	X
18M	X
17D	X
17M	X
16D	X
16M	X
15D	X
15M	X
14D	70%
14M	72%
13D	75%
13M	77%
12D	78%
12M	80%
11D	83%
11M	85%
21M	85%
21D	82%
22M	80%
22D	78%
23M	75%
23D	73%
24M	70%
24D	68%
25M	X
25D	X
26M	X
26D	X
27M	X
27D	X
28M	X
28D	X
38D	X
38M	X
37D	X
37M	X
36D	X
36M	X
35D	65%
35M	68%
34D	70%
34M	73%
33D	75%
33M	77%
32D	80%

32M	83%
31D	85%
31M	85%
41M	85%
41D	85%
42M	82%
42D	80%
43M	78%
43D	75%
44M	73%
44D	70%
45M	68%
45D	65%
46M	60%
46D	58%
47M	X
47D	X
48M	X
48D	X
