# Supplementary material for: Advances in Periodontal Diagnostics: Application of MultiModal Language Models in Visual Interpretation of Panoramic Radiographs
Source: Diagnostics (Basel). 2025 Jul 23;15(15):1851. doi: 10.3390/diagnostics15151851 (PMC12345756; doi:10.3390/diagnostics15151851)
Supplement: Supplementary file 1 [file diagnostics-15-01851-s001.zip › ChatGPT_Reports.zip!/o1.zip!/3_ChatGPT_o1.pdf]

1. Tooth Count

Table 1. Evaluation of the Presence of Teeth

Tooth no.	Tooth Presence
18	0
17	1
16	1
15	1
14	1
13	1
12	1
11	1
21	1
22	1
23	1
24	1
25	1
26	1
27	1
28	0
38	0
37	0
36	1
35	1
34	1
33	1
32	1
31	1
41	1
42	1
43	1
44	1
45	1
46	1
47	0
48	0

Table 2. Evaluation of the Remaining Bone Height (RBH)

Approximal surfaces	Remaining bone height
18D	N/A
18M	N/A
17D	70%
17M	70%
16D	70%
16M	70%
15D	75%
15M	75%
14D	75%
14M	75%
13D	80%
13M	80%
12D	80%
12M	80%
11D	80%
11M	80%
21M	80%
21D	80%
22M	80%
22D	80%
23M	80%
23D	80%
24M	75%
24D	75%
25M	75%
25D	75%
26M	70%
26D	70%
27M	70%
27D	70%
28M	N/A
28D	N/A
38D	N/A
38M	N/A
37D	N/A
37M	N/A
36D	60%
36M	60%

Approximal surfaces	Remaining bone height
35D	60%
35M	60%
34D	60%
34M	60%
33D	55%
33M	55%
32D	55%
32M	55%
31D	55%
31M	55%
41M	55%
41D	55%
42M	55%
42D	55%
43M	55%
43D	55%
44M	60%
44D	60%
45M	60%
45D	60%
46M	60%
46D	60%
47M	N/A
47D	N/A
48M	N/A
48D	N/A
