# Supplementary material for: Advances in Periodontal Diagnostics: Application of MultiModal Language Models in Visual Interpretation of Panoramic Radiographs
Source: Diagnostics (Basel). 2025 Jul 23;15(15):1851. doi: 10.3390/diagnostics15151851 (PMC12345756; doi:10.3390/diagnostics15151851)
Supplement: Supplementary file 1 [file diagnostics-15-01851-s001.zip › ChatGPT_Reports.zip!/o1.zip!/4_ChatGPT_o1.pdf]

Table 1. Evaluation of the Presence of Teeth

Tooth no.	Tooth Presence
18	0
17	1
16	1
15	0
14	0
13	0
12	0
11	0
21	0
22	0
23	0
24	0
25	0
26	0
27	0
28	0
38	0
37	1
36	1
35	0
34	0
33	1
32	1
31	1
41	1
42	1
43	1
44	0
45	0
46	1
47	0
48	0

Table 2. Evaluation of the Remaining Bone Height (RBH)

Approximal surfaces	Remaining bone height
18D	N/A
18M	N/A
17D	40%
17M	35%
16D	30%
16M	35%
15D	N/A
15M	N/A
14D	N/A
14M	N/A
13D	N/A
13M	N/A
12D	N/A
12M	N/A
11D	N/A
11M	N/A
21M	N/A
21D	N/A
22M	N/A
22D	N/A
23M	N/A
23D	N/A
24M	N/A
24D	N/A
25M	N/A
25D	N/A
26M	N/A
26D	N/A
27M	N/A
27D	N/A
28M	N/A
28D	N/A
38D	N/A
38M	N/A
37D	45%
37M	40%
36D	50%
36M	45%

Approximal surfaces	Remaining bone height
35D	N/A
35M	N/A
34D	N/A
34M	N/A
33D	60%
33M	60%
32D	70%
32M	70%
31D	75%
31M	75%
41M	75%
41D	75%
42M	70%
42D	70%
43M	60%
43D	60%
44M	N/A
44D	N/A
45M	N/A
45D	N/A
46M	40%
46D	35%
47M	N/A
47D	N/A
48M	N/A
48D	N/A
