# Supplementary material for: Advances in Periodontal Diagnostics: Application of MultiModal Language Models in Visual Interpretation of Panoramic Radiographs
Source: Diagnostics (Basel). 2025 Jul 23;15(15):1851. doi: 10.3390/diagnostics15151851 (PMC12345756; doi:10.3390/diagnostics15151851)
Supplement: Supplementary file 1 [file diagnostics-15-01851-s001.zip › ChatGPT_Reports.zip!/o1.zip!/5_ChatGPT_o1.pdf]

Table no. 1 – Evaluation of the presence of teeth

Tooth no.	Tooth Presence
18	0
17	0
16	0
15	0
14	0
13	0
12	0
11	0
21	0
22	0
23	0
24	0
25	0
26	0
27	0
28	0
38	0
37	1
36	1
35	1
34	0
33	0
32	0
31	0
41	0
42	0
43	0
44	0
45	1
46	1
47	1
48	0

Table no. 2 – Evaluation of the remaining bone height (RBH)

RBH is expressed here as a percentage (%). Surfaces with no tooth present are indicated as "N/A."

Approximal surfaces	Remaining bone height
18D	N/A
18M	N/A
17D	N/A
17M	N/A
16D	N/A
16M	N/A
15D	N/A
15M	N/A
14D	N/A
14M	N/A
13D	N/A
13M	N/A
12D	N/A
12M	N/A
11D	N/A
11M	N/A
21M	N/A
21D	N/A
22M	N/A
22D	N/A
23M	N/A
23D	N/A
24M	N/A
24D	N/A
25M	N/A
25D	N/A
26M	N/A
26D	N/A
27M	N/A
27D	N/A
28M	N/A
28D	N/A
38D	N/A
38M	N/A

Approximal surfaces	Remaining bone height
37D	50%
37M	55%
36D	55%
36M	60%
35D	65%
35M	65%
34D	N/A
34M	N/A
33D	N/A
33M	N/A
32D	N/A
32M	N/A
31D	N/A
31M	N/A
41M	N/A
41D	N/A
42M	N/A
42D	N/A
43M	N/A
43D	N/A
44M	N/A
44D	N/A
45M	70%
45D	70%
46M	60%
46D	55%
47M	55%
47D	50%
48M	N/A
48D	N/A
