# Supplementary material for: Advances in Periodontal Diagnostics: Application of MultiModal Language Models in Visual Interpretation of Panoramic Radiographs
Source: Diagnostics (Basel). 2025 Jul 23;15(15):1851. doi: 10.3390/diagnostics15151851 (PMC12345756; doi:10.3390/diagnostics15151851)
Supplement: Supplementary file 1 [file diagnostics-15-01851-s001.zip › ChatGPT_Reports.zip!/o1.zip!/6_ChatGPT_o1.pdf]

Table No. 1 – Evaluation of the Presence of Teeth

Tooth no.	Tooth Presence
18	1
17	1
16	1
15	1
14	1
13	1
12	1
11	1
21	1
22	1
23	1
24	1
25	1
26	1
27	1
28	1
38	1
37	1
36	1
35	1
34	1
33	1
32	1
31	1
41	1
42	1
43	1
44	1
45	1
46	1
47	1
48	1

Table No. 2 – Evaluation of the Remaining Bone Height (RBH)

Note: In this case, all teeth are present. If a tooth had been absent, the corresponding surfaces would be marked “X.”

**Approximal surfaces**	**Remaining bone height**
18D	80%
18M	80%
17D	85%
17M	85%
16D	88%
16M	88%
15D	90%
15M	90%
14D	90%
14M	90%
13D	92%
13M	92%
12D	92%
12M	92%
11D	92%
11M	92%
21M	92%
21D	92%
22M	92%
22D	92%
23M	92%
23D	92%
24M	90%
24D	90%
25M	90%
25D	90%
26M	88%
26D	88%
27M	85%
27D	85%
28M	80%
28D	80%
38D	80%
38M	80%

Approximal surfaces	Remaining bone height
37D	85%
37M	85%
36D	88%
36M	88%
35D	90%
35M	90%
34D	90%
34M	90%
33D	92%
33M	92%
32D	92%
32M	92%
31D	92%
31M	92%
41M	92%
41D	92%
42M	92%
42D	92%
43M	92%
43D	92%
44M	90%
44D	90%
45M	90%
45D	90%
46M	88%
46D	88%
47M	85%
47D	85%
48M	80%
48D	80%
