# Supplementary material for: Advances in Periodontal Diagnostics: Application of MultiModal Language Models in Visual Interpretation of Panoramic Radiographs
Source: Diagnostics (Basel). 2025 Jul 23;15(15):1851. doi: 10.3390/diagnostics15151851 (PMC12345756; doi:10.3390/diagnostics15151851)
Supplement: Supplementary file 1 [file diagnostics-15-01851-s001.zip › ChatGPT_Reports.zip!/o1.zip!/8_ChatGPT_o1.pdf]

2. Table No. 1 – Evaluation of the Presence of Teeth

Tooth no.	Tooth Presence
18	1
17	1
16	1
15	1
14	1
13	1
12	1
11	1
21	1
22	1
23	1
24	1
25	1
26	1
27	1
28	1
38	1
37	1
36	1
35	1
34	1
33	1
32	1
31	1
41	1
42	1
43	1
44	1
45	1
46	1
47	1
48	1

3. Table No. 2 – Evaluation of the Remaining Bone Height (RBH)

Definition of RBH:

RBH (%)=(Distance from marginal bone crest to radiographic root apex)(Distance from CEJ to radiographic root apex)×100%\text{RBH (\%)} = \frac{\text{(Distance from marginal bone crest to radiographic root apex)}}{\text{(Distance from CEJ to radiographic root apex)}} \times 100\%RBH (%)=(Distance from CEJ to radiographic root apex)(Distance from marginal bone crest to radiographic root apex)×100%

- **M** = Mesial surface
- **D** = Distal surface
- **X** = Not applicable (tooth absent)

Because **all teeth are present**, no cells in the table are marked with "X."
Below are sample percentage values based on visual approximation of the bone crest level. (In clinical practice, these should be measured carefully and compared bilaterally.)

**Approximal surfaces**	**Remaining bone height**
18D	85%
18M	85%
17D	90%
17M	90%
16D	90%
16M	90%
15D	92%
15M	92%
14D	93%
14M	93%
13D	95%
13M	95%
12D	95%
12M	95%
11D	95%
11M	95%
21M	95%
21D	95%
22M	95%
22D	95%
23M	95%
23D	95%
24M	93%

**Approximal surfaces**	**Remaining bone height**
24D	93%
25M	92%
25D	92%
26M	90%
26D	90%
27M	88%
27D	88%
28M	85%
28D	85%
38D	80%
38M	80%
37D	90%
37M	90%
36D	92%
36M	92%
35D	93%
35M	93%
34D	94%
34M	94%
33D	95%
33M	95%
32D	95%
32M	95%
31D	95%
31M	95%
41M	95%
41D	95%
42M	95%
42D	95%
43M	95%
43D	95%
44M	93%
44D	93%
45M	92%
45D	92%
46M	90%
46D	90%

**Approximal surfaces**	**Remaining bone height**
47M	88%
47D	88%
48M	80%
48D	80%
