# Supplementary material for: Advances in Periodontal Diagnostics: Application of MultiModal Language Models in Visual Interpretation of Panoramic Radiographs
Source: Diagnostics (Basel). 2025 Jul 23;15(15):1851. doi: 10.3390/diagnostics15151851 (PMC12345756; doi:10.3390/diagnostics15151851)
Supplement: Supplementary file 1 [file diagnostics-15-01851-s001.zip › ChatGPT_Reports.zip!/o3.zip!/10_ChatGPT_o3.pdf]

Radiological Report – Panoramic Radiograph

Date: 2025-04-22

Evaluation of the presence of teeth

Tooth no.	Tooth Presence
18	0
17	1
16	1
15	1
14	1
13	1
12	1
11	1
21	1
22	1
23	1
24	1
25	1
26	1
27	1
28	0
38	0
37	1
36	1
35	1

34	1
33	1
32	1
31	1
41	1
42	1
43	1
44	1
45	1
46	1
47	1
48	0

Evaluation of the remaining bone height (RBH)

Approximal surfaces	Remaining bone height
18D	X
18M	X
17D	85%
17M	85%
16D	90%
16M	90%
15D	95%
15M	95%
14D	95%
14M	95%
13D	100%

13M	100%
12D	100%
12M	100%
11D	100%
11M	100%
21M	100%
21D	100%
22M	100%
22D	100%
23M	100%
23D	100%
24M	95%
24D	95%
25M	95%
25D	95%
26M	90%
26D	90%
27M	85%
27D	85%
28M	X
28D	X
38D	X
38M	X
37D	85%
37M	85%
36D	90%

36M	90%
35D	95%
35M	95%
34D	95%
34M	95%
33D	100%
33M	100%
32D	100%
32M	100%
31D	100%
31M	100%
41M	100%
41D	100%
42M	100%
42D	100%
43M	100%
43D	100%
44M	95%
44D	95%
45M	95%
45D	95%
46M	90%
46D	90%
47M	85%
47D	85%
48M	X

48D X
