# Supplementary material for: Advances in Periodontal Diagnostics: Application of MultiModal Language Models in Visual Interpretation of Panoramic Radiographs
Source: Diagnostics (Basel). 2025 Jul 23;15(15):1851. doi: 10.3390/diagnostics15151851 (PMC12345756; doi:10.3390/diagnostics15151851)
Supplement: Supplementary file 1 [file diagnostics-15-01851-s001.zip › ChatGPT_Reports.zip!/o3.zip!/1_ChatGPT_o3.pdf]

Radiological Report – Panoramic Radiograph

This report summarizes the evaluation of tooth presence and remaining bone height (RBH) on the provided panoramic radiograph (file CH1.png). All tooth numbers follow the FDI two-digit notation.

Table 1 – Evaluation of the presence of teeth

Tooth no.	Tooth presence
18	0
17	1
16	1
15	1
14	1
13	1
12	1
11	1
21	1
22	1
23	1
24	1
25	1
26	1
27	1
28	0
38	1
37	1
36	1
35	1
34	1
33	1
32	1
31	1
41	1
42	1
43	1
44	1
45	1
46	1
47	1
48	1

Table 2 – Evaluation of the remaining bone height (RBH)

Approximal surface	Remaining bone height (%)
18D	X
18M	X
17D	95

17M	95
16D	95
16M	95
15D	95
15M	95
14D	95
14M	95
13D	95
13M	95
12D	95
12M	95
11D	95
11M	95
21M	95
21D	95
22M	95
22D	95
23M	95
23D	95
24M	95
24D	95
25M	95
25D	95
26M	95
26D	95
27M	95
27D	95
28M	X
28D	X
38D	95
38M	95
37D	95
37M	95
36D	95
36M	95
35D	95
35M	95
34D	95
34M	95
33D	95
33M	95
32D	95
32M	95
31D	95
31M	95
41M	95
41D	95
42M	95

42D	95
43M	95
43D	95
44M	95
44D	95
45M	95
45D	95
46M	95
46D	95
47M	95
47D	95
48M	95
48D	95
