# Supplementary material for: Advances in Periodontal Diagnostics: Application of MultiModal Language Models in Visual Interpretation of Panoramic Radiographs
Source: Diagnostics (Basel). 2025 Jul 23;15(15):1851. doi: 10.3390/diagnostics15151851 (PMC12345756; doi:10.3390/diagnostics15151851)
Supplement: Supplementary file 1 [file diagnostics-15-01851-s001.zip › ChatGPT_Reports.zip!/o3.zip!/2_ChatGPT_o3.pdf]

Panoramic Radiograph Report

Date of analysis: 2025-04-22

Radiological evaluation of tooth presence and remaining bone height (RBH).

Evaluation of the presence of teeth

Tooth no.	Tooth Presence
18	0
17	0
16	1
15	1
14	1
13	1
12	1
11	1
21	1
22	1
23	1
24	1
25	1
26	1
27	0
28	0
38	0
37	0
36	0
35	0

34	0
33	0
32	1
31	1
41	1
42	1
43	1
44	1
45	0
46	0
47	0
48	0

Evaluation of the remaining bone height (RBH)

Approximal surfaces	Remaining bone height (%)
18D	X
18M	X
17D	X
17M	X
16D	70
16M	70
15D	70
15M	70
14D	70
14M	70
13D	70

13M	70
12D	70
12M	70
11D	70
11M	70
21M	70
21D	70
22M	70
22D	70
23M	70
23D	70
24M	70
24D	70
25M	70
25D	70
26M	70
26D	70
27M	X
27D	X
28M	X
28D	X
38D	X
38M	X
37D	X
37M	X
36D	X

36M	X
35D	X
35M	X
34D	X
34M	X
33D	X
33M	X
32D	70
32M	70
31D	70
31M	70
41M	70
41D	70
42M	70
42D	70
43M	70
43D	70
44M	70
44D	70
45M	X
45D	X
46M	X
46D	X
47M	X
47D	X
48M	X

48D X
