# Supplementary material for: Advances in Periodontal Diagnostics: Application of MultiModal Language Models in Visual Interpretation of Panoramic Radiographs
Source: Diagnostics (Basel). 2025 Jul 23;15(15):1851. doi: 10.3390/diagnostics15151851 (PMC12345756; doi:10.3390/diagnostics15151851)
Supplement: Supplementary file 1 [file diagnostics-15-01851-s001.zip › ChatGPT_Reports.zip!/o3.zip!/3_ChatGPT_o3.pdf]

Panoramic Radiograph Report

Tooth no.	Tooth Presence
18	0
17	0
16	0
15	0
14	0
13	1
12	1
11	1
21	1
22	1
23	1
24	0
25	0
26	0
27	0
28	0
38	0
37	0
36	1
35	1
34	1
33	1
32	1
31	1
41	1
42	1
43	1
44	1
45	1
46	1
47	0
48	0
Approximal surfaces	Remaining bone height (%)
18D	X
18M	X
17D	X
17M	X
16D	X
16M	X
15D	X
15M	X
14D	X
14M	X
13D	85

13M	85
12D	85
12M	85
11D	85
11M	85
21M	85
21D	85
22M	85
22D	85
23M	85
23D	85
24M	X
24D	X
25M	X
25D	X
26M	X
26D	X
27M	X
27D	X
28M	X
28D	X
38D	X
38M	X
37D	X
37M	X
36D	85
36M	85
35D	85
35M	85
34D	85
34M	85
33D	85
33M	85
32D	85
32M	85
31D	85
31M	85
41M	85
41D	85
42M	85
42D	85
43M	85
43D	85
44M	85
44D	85
45M	85
45D	85
46M	85

46D	85
47M	X
47D	X
48M	X
48D	X
