# Supplementary material for: Advances in Periodontal Diagnostics: Application of MultiModal Language Models in Visual Interpretation of Panoramic Radiographs
Source: Diagnostics (Basel). 2025 Jul 23;15(15):1851. doi: 10.3390/diagnostics15151851 (PMC12345756; doi:10.3390/diagnostics15151851)
Supplement: Supplementary file 1 [file diagnostics-15-01851-s001.zip › ChatGPT_Reports.zip!/o3.zip!/4_ChatGPT_o3.pdf]

Radiological Report – Panoramic Radiograph

Modality: Panoramic radiograph
Note: Tooth presence and remaining bone height (RBH) were assessed visually. RBH values are approximate (%). “X” denotes tooth absence.

Table 1 – Evaluation of the presence of teeth

Tooth no.	Tooth presence
18	0
17	0
16	0
15	1
14	1
13	1
12	0
11	0
21	0
22	0
23	0
24	0
25	0
26	0
27	0
28	0
38	0
37	1
36	0

35	0
34	0
33	1
32	1
31	1
41	1
42	1
43	1
44	1
45	0
46	0
47	0
48	0

Table 2 – Evaluation of the remaining bone height (RBH)

Approximal surfaces	Remaining bone height (%)
18D	X
18M	X
17D	X
17M	X
16D	X
16M	X
15D	40
15M	40
14D	40
14M	40
13D	40
13M	40
12D	X
12M	X
11D	X
11M	X
21M	X
21D	X
22M	X
22D	X
23M	X
23D	X
24M	X
24D	X

25M	X
25D	X
26M	X
26D	X
27M	X
27D	X
28M	X
28D	X
38D	X
38M	X
37D	70
37M	70
36D	X
36M	X
35D	X
35M	X
34D	X
34M	X
33D	60
33M	60
32D	60
32M	60
31D	60
31M	60
41M	60
41D	60

42M	60
42D	60
43M	60
43D	60
44M	65
44D	65
45M	X
45D	X
46M	X
46D	X
47M	X
47D	X
48M	X
48D	X
