# Supplementary material for: Advances in Periodontal Diagnostics: Application of MultiModal Language Models in Visual Interpretation of Panoramic Radiographs
Source: Diagnostics (Basel). 2025 Jul 23;15(15):1851. doi: 10.3390/diagnostics15151851 (PMC12345756; doi:10.3390/diagnostics15151851)
Supplement: Supplementary file 1 [file diagnostics-15-01851-s001.zip › ChatGPT_Reports.zip!/o3.zip!/6_ChatGPT_o3.pdf]

Panoramic Radiograph Report

Patient panoramic radiograph analysis – tooth presence and remaining bone height (RBH).

Tooth no.	Tooth Presence
18	0
17	1
16	1
15	1
14	1
13	1
12	1
11	1
21	1
22	1
23	1
24	1
25	1
26	1
27	1
28	0
38	0
37	1
36	1
35	1
34	1
33	1

32	1
31	1
41	1
42	1
43	1
44	1
45	1
46	1
47	1
48	0

Approximal surfaces	Remaining bone height (%)
18D	X
18M	X
17D	90
17M	90
16D	90
16M	90
15D	90
15M	90
14D	90
14M	90
13D	90
13M	90
12D	90
12M	90

11D	90
11M	90
21M	90
21D	90
22M	90
22D	90
23M	90
23D	90
24M	90
24D	90
25M	90
25D	90
26M	90
26D	90
27M	90
27D	90
28M	X
28D	X
38D	X
38M	X
37D	90
37M	90
36D	90
36M	90
35D	90
35M	90

34D	90
34M	90
33D	90
33M	90
32D	90
32M	90
31D	90
31M	90
41M	90
41D	90
42M	90
42D	90
43M	90
43D	90
44M	90
44D	90
45M	90
45D	90
46M	90
46D	90
47M	90
47D	90
48M	X
48D	X
