# Supplementary material for: Advances in Periodontal Diagnostics: Application of MultiModal Language Models in Visual Interpretation of Panoramic Radiographs
Source: Diagnostics (Basel). 2025 Jul 23;15(15):1851. doi: 10.3390/diagnostics15151851 (PMC12345756; doi:10.3390/diagnostics15151851)
Supplement: Supplementary file 1 [file diagnostics-15-01851-s001.zip › ChatGPT_Reports.zip!/o3.zip!/7_ChatGPT_o3.pdf]

Radiological Report – Panoramic Radiograph Analysis

Table 1 – Evaluation of the presence of teeth

Tooth no.	Tooth presence
18	0
17	1
16	1
15	1
14	1
13	1
12	1
11	1
21	1
22	1
23	1
24	1
25	1
26	1
27	1
28	0
38	1
37	1
36	1
35	1
34	1
33	1

32	1
31	1
41	1
42	1
43	1
44	1
45	1
46	1
47	1
48	1

Table 2 – Evaluation of the remaining bone height (RBH)

Approximal surface	Remaining bone height (%)
18D	X
18M	X
17D	85
17M	85
16D	85
16M	85
15D	90
15M	90
14D	90
14M	90
13D	95
13M	95
12D	95

12M	95
11D	95
11M	95
21M	95
21D	95
22M	95
22D	95
23M	95
23D	95
24M	90
24D	90
25M	90
25D	90
26M	85
26D	85
27M	85
27D	85
28M	X
28D	X
38D	85
38M	85
37D	85
37M	85
36D	85
36M	85
35D	90

35M	90
34D	90
34M	90
33D	95
33M	95
32D	95
32M	95
31D	95
31M	95
41M	95
41D	95
42M	95
42D	95
43M	95
43D	95
44M	90
44D	90
45M	90
45D	90
46M	85
46D	85
47M	85
47D	85
48M	85
48D	85
