# Supplementary material for: Advances in Periodontal Diagnostics: Application of MultiModal Language Models in Visual Interpretation of Panoramic Radiographs
Source: Diagnostics (Basel). 2025 Jul 23;15(15):1851. doi: 10.3390/diagnostics15151851 (PMC12345756; doi:10.3390/diagnostics15151851)
Supplement: Supplementary file 1 [file diagnostics-15-01851-s001.zip › ChatGPT_Reports.zip!/o3.zip!/8_ChatGPT_o3.pdf]

# Panoramic Radiograph Report

## Evaluation of the presence of teeth

| Tooth no. | Tooth Presence |
|---|---|
| 18 | 0 |
| 17 | 1 |
| 16 | 1 |
| 15 | 1 |
| 14 | 1 |
| 13 | 1 |
| 12 | 1 |
| 11 | 1 |
| 21 | 1 |
| 22 | 1 |
| 23 | 1 |
| 24 | 1 |
| 25 | 1 |
| 26 | 1 |
| 27 | 1 |
| 28 | 0 |
| 38 | 0 |
| 37 | 1 |
| 36 | 1 |
| 35 | 1 |
| 34 | 1 |
| 33 | 1 |

| | |
|---|---|
| 32 | 1 |
| 31 | 1 |
| 41 | 1 |
| 42 | 1 |
| 43 | 1 |
| 44 | 1 |
| 45 | 1 |
| 46 | 1 |
| 47 | 1 |
| 48 | 0 |

## Evaluation of the remaining bone height (RBH)

| Approximal surfaces | Remaining bone height |
|---|---|
| 18D | X |
| 18M | X |
| 17D | 90% |
| 17M | 90% |
| 16D | 90% |
| 16M | 90% |
| 15D | 90% |
| 15M | 90% |
| 14D | 90% |
| 14M | 90% |
| 13D | 90% |
| 13M | 90% |
| 12D | 90% |
| 12M | 90% |
| 11D | 90% |
| 11M | 90% |
| 21M | 90% |
| 21D | 90% |
| 22M | 90% |
| 22D | 90% |
| 23M | 90% |
| 23D | 90% |
| 24M | 90% |
| 24D | 90% |

| | |
|---|---|
| 25M | 90% |
| 25D | 90% |
| 26M | 90% |
| 26D | 90% |
| 27M | 90% |
| 27D | 90% |
| 28M | X |
| 28D | X |
| 38D | X |
| 38M | X |
| 37D | 90% |
| 37M | 90% |
| 36D | 90% |
| 36M | 90% |
| 35D | 90% |
| 35M | 90% |
| 34D | 90% |
| 34M | 90% |
| 33D | 90% |
| 33M | 90% |
| 32D | 90% |
| 32M | 90% |
| 31D | 90% |
| 31M | 90% |
| 41M | 90% |
| 41D | 90% |

| | |
|---|---|
| 42M | 90% |
| 42D | 90% |
| 43M | 90% |
| 43D | 90% |
| 44M | 90% |
| 44D | 90% |
| 45M | 90% |
| 45D | 90% |
| 46M | 90% |
| 46D | 90% |
| 47M | 90% |
| 47D | 90% |
| 48M | X |
| 48D | X |
