# Supplementary material for: Advances in Periodontal Diagnostics: Application of MultiModal Language Models in Visual Interpretation of Panoramic Radiographs
Source: Diagnostics (Basel). 2025 Jul 23;15(15):1851. doi: 10.3390/diagnostics15151851 (PMC12345756; doi:10.3390/diagnostics15151851)
Supplement: Supplementary file 1 [file diagnostics-15-01851-s001.zip › ChatGPT_Reports.zip!/o3.zip!/9_ChatGPT_o3.pdf]

Panoramic Radiograph Report

Table 1 – Evaluation of the Presence of Teeth

Tooth no.	Tooth Presence
18	0
17	0
16	0
15	0
14	0
13	1
12	1
11	1
21	1
22	1
23	1
24	0
25	0
26	0
27	0
28	0
38	0
37	0
36	0
35	0
34	0
33	1

32	1
31	1
41	1
42	1
43	1
44	0
45	0
46	0
47	0
48	0

Table 2 – Evaluation of the Remaining Bone Height (RBH)

Approximal surfaces	Remaining bone height (%)
18D	X
18M	X
17D	X
17M	X
16D	X
16M	X
15D	X
15M	X
14D	X
14M	X
13D	75
13M	80
12D	82

12M	78
11D	85
11M	85
21M	85
21D	85
22M	78
22D	82
23M	80
23D	75
24M	X
24D	X
25M	X
25D	X
26M	X
26D	X
27M	X
27D	X
28M	X
28D	X
38D	X
38M	X
37D	X
37M	X
36D	X
36M	X
35D	X

35M	X
34D	X
34M	X
33D	70
33M	70
32D	68
32M	68
31D	65
31M	65
41M	65
41D	65
42M	68
42D	68
43M	70
43D	70
44M	X
44D	X
45M	X
45D	X
46M	X
46D	X
47M	X
47D	X
48M	X
48D	X
