# Supplementary material for: Advances in Periodontal Diagnostics: Application of MultiModal Language Models in Visual Interpretation of Panoramic Radiographs
Source: Diagnostics (Basel). 2025 Jul 23;15(15):1851. doi: 10.3390/diagnostics15151851 (PMC12345756; doi:10.3390/diagnostics15151851)
Supplement: Supplementary file 1 [file diagnostics-15-01851-s001.zip › ChatGPT_Reports.zip!/o4-mini-high.zip!/10_ChatGPT_o4-mini-high.pdf]

Radiological Report

1. Evaluation of the presence of teeth

Tooth no.	Tooth Presence
18	1
17	1
16	1
15	1
14	1
13	1
12	1
11	1
21	1
22	1
23	1
24	1
25	1
26	1
27	1
28	1
38	1
37	1
36	1
35	1
34	1
33	1

32	1
31	1
41	1
42	1
43	1
44	1
45	1
46	1
47	1
48	1

2. Evaluation of the remaining bone height (RBH)

Approximal surfaces	Remaining bone height (%)
18D	90%
18M	90%
17D	90%
17M	90%
16D	90%
16M	90%
15D	90%
15M	90%
14D	90%
14M	90%
13D	90%
13M	90%
12D	90%

12M	90%
11D	90%
11M	90%
21M	90%
21D	90%
22M	90%
22D	90%
23M	90%
23D	90%
24M	90%
24D	90%
25M	90%
25D	90%
26M	90%
26D	90%
27M	90%
27D	90%
28M	90%
28D	90%
38D	90%
38M	90%
37D	90%
37M	90%
36D	90%
36M	90%
35D	90%

35M	90%
34D	90%
34M	90%
33D	90%
33M	90%
32D	90%
32M	90%
31D	90%
31M	90%
41M	90%
41D	90%
42M	90%
42D	90%
43M	90%
43D	90%
44M	90%
44D	90%
45M	90%
45D	90%
46M	90%
46D	90%
47M	90%
47D	90%
48M	90%
48D	90%
