# Supplementary material for: Advances in Periodontal Diagnostics: Application of MultiModal Language Models in Visual Interpretation of Panoramic Radiographs
Source: Diagnostics (Basel). 2025 Jul 23;15(15):1851. doi: 10.3390/diagnostics15151851 (PMC12345756; doi:10.3390/diagnostics15151851)
Supplement: Supplementary file 1 [file diagnostics-15-01851-s001.zip › ChatGPT_Reports.zip!/o4-mini-high.zip!/2_ChatGPT_o4-mini-high.pdf]

Radiological Report

Table 1 - Evaluation of the presence of teeth

Tooth no.	Tooth Presence
18	0
17	1
16	1
15	1
14	1
13	1
12	1
11	1
21	1
22	1
23	1
24	1
25	1
26	1
27	1
28	0
38	0
37	0
36	0
35	0
34	0
33	1

32	1
31	1
41	1
42	1
43	1
44	0
45	0
46	0
47	0
48	0

Table 2 - Evaluation of the remaining bone height (RBH)

Approximal surfaces	Remaining bone height
18D	X
18M	X
17D	90%
17M	90%
16D	90%
16M	90%
15D	90%
15M	90%
14D	90%
14M	90%
13D	90%
13M	90%
12D	90%
12M	90%
11D	90%
11M	90%
21M	90%
21D	90%
22M	90%
22D	90%
23M	90%
23D	90%
24M	90%
24D	90%

25M	90%
25D	90%
26M	90%
26D	90%
27M	90%
27D	90%
28M	X
28D	X
38D	X
38M	X
37D	X
37M	X
36D	X
36M	X
35D	X
35M	X
34D	X
34M	X
33D	90%
33M	90%
32D	90%
32M	90%
31D	90%
31M	90%
41M	90%
41D	90%

42M	90%
42D	90%
43M	90%
43D	90%
44M	X
44D	X
45M	X
45D	X
46M	X
46D	X
47M	X
47D	X
48M	X
48D	X
