# Supplementary material for: Advances in Periodontal Diagnostics: Application of MultiModal Language Models in Visual Interpretation of Panoramic Radiographs
Source: Diagnostics (Basel). 2025 Jul 23;15(15):1851. doi: 10.3390/diagnostics15151851 (PMC12345756; doi:10.3390/diagnostics15151851)
Supplement: Supplementary file 1 [file diagnostics-15-01851-s001.zip › ChatGPT_Reports.zip!/o4-mini-high.zip!/5_ChatGPT_o4-mini-high.pdf]

Radiological Report

Table no. 1 - Evaluation of the presence of teeth

Tooth no.	Tooth Presence
18	0
17	1
16	0
15	0
14	0
13	0
12	0
11	0
21	0
22	0
23	0
24	0
25	0
26	0
27	1
28	0
38	0
37	0
36	1
35	0
34	0
33	1

32	1
31	1
41	1
42	1
43	0
44	0
45	0
46	0
47	0
48	0

Table no. 2 - Evaluation of the remaining bone height (RBH)

Approximal surfaces	Remaining bone height
18D	X
18M	X
17D	50%
17M	45%
16D	X
16M	X
15D	X
15M	X
14D	X
14M	X
13D	X
13M	X
12D	X

12M	X
11D	X
11M	X
21M	X
21D	X
22M	X
22D	X
23M	X
23D	X
24M	X
24D	X
25M	X
25D	X
26M	X
26D	X
27M	50%
27D	55%
28M	X
28D	X
38D	X
38M	X
37D	X
37M	X
36D	55%
36M	60%
35D	X

35M	X
34D	X
34M	X
33D	65%
33M	70%
32D	75%
32M	80%
31D	75%
31M	80%
41M	80%
41D	75%
42M	80%
42D	75%
43M	X
43D	X
44M	X
44D	X
45M	X
45D	X
46M	X
46D	X
47M	X
47D	X
48M	X
48D	X
