# Supplementary material for: Advances in Periodontal Diagnostics: Application of MultiModal Language Models in Visual Interpretation of Panoramic Radiographs
Source: Diagnostics (Basel). 2025 Jul 23;15(15):1851. doi: 10.3390/diagnostics15151851 (PMC12345756; doi:10.3390/diagnostics15151851)
Supplement: Supplementary file 1 [file diagnostics-15-01851-s001.zip › ChatGPT_Reports.zip!/o4-mini-high.zip!/6_ChatGPT_o4-mini-high.pdf]

Panoramic Radiograph Analysis Report

1. Tooth Count

Number of present teeth: 30

Evaluation of the presence of teeth

Tooth no.	Tooth Presence
18	0
17	1
16	1
15	1
14	1
13	1
12	1
11	1
21	1
22	1
23	1
24	1
25	1
26	1
27	1
28	0
38	1
37	1
36	1
35	1

34	1
33	1
32	1
31	1
41	1
42	1
43	1
44	1
45	1
46	1
47	1
48	1

2. Evaluation of the remaining bone height (RBH)

RBH is calculated as the percentage ratio of the distance from the marginal bone crest to the root apex over the distance from the cemento-enamel junction (CEJ) to the root apex. "X" indicates missing tooth surfaces.

Approximal surfaces	Remaining bone height
18D	X
18M	X
17D	90%
17M	90%
16D	90%
16M	90%
15D	90%
15M	90%
14D	90%

14M	90%
13D	90%
13M	90%
12D	90%
12M	90%
11D	90%
11M	90%
21M	90%
21D	90%
22M	90%
22D	90%
23M	90%
23D	90%
24M	90%
24D	90%
25M	90%
25D	90%
26M	90%
26D	90%
27M	90%
27D	90%
28M	X
28D	X
38D	90%
38M	90%
37D	90%

37M	90%
36D	90%
36M	90%
35D	90%
35M	90%
34D	90%
34M	90%
33D	90%
33M	90%
32D	90%
32M	90%
31D	90%
31M	90%
41M	90%
41D	90%
42M	90%
42D	90%
43M	90%
43D	90%
44M	90%
44D	90%
45M	90%
45D	90%
46M	90%
46D	90%
47M	90%

47D	90%
48M	90%
48D	90%
