# Supplementary material for: Advances in Periodontal Diagnostics: Application of MultiModal Language Models in Visual Interpretation of Panoramic Radiographs
Source: Diagnostics (Basel). 2025 Jul 23;15(15):1851. doi: 10.3390/diagnostics15151851 (PMC12345756; doi:10.3390/diagnostics15151851)
Supplement: Supplementary file 1 [file diagnostics-15-01851-s001.zip › ChatGPT_Reports.zip!/o4-mini-high.zip!/7_ChatGPT_o4-mini-high.pdf]

## Radiological Report: Panoramic Radiograph Analysis

### 1. Tooth Count

Number of present teeth: 32

#### Evaluation of the presence of teeth

| Tooth no. | Tooth Presence |
|---|---|
| 18 | 1 |
| 17 | 1 |
| 16 | 1 |
| 15 | 1 |
| 14 | 1 |
| 13 | 1 |
| 12 | 1 |
| 11 | 1 |
| 21 | 1 |
| 22 | 1 |
| 23 | 1 |
| 24 | 1 |
| 25 | 1 |
| 26 | 1 |
| 27 | 1 |
| 28 | 1 |
| 38 | 1 |
| 37 | 1 |
| 36 | 1 |
| 35 | 1 |
| 34 | 1 |
| 33 | 1 |
| 32 | 1 |
| 31 | 1 |
| 41 | 1 |
| 42 | 1 |
| 43 | 1 |
| 44 | 1 |
| 45 | 1 |
| 46 | 1 |
| 47 | 1 |
| 48 | 1 |

### 2. Remaining Bone Height (RBH)

RBH values are presented as the percentage of the distance from the marginal bone crest to the root apex relative to the distance from the CEJ to the root apex. All measured sites appear to have healthy bone levels with RBH approximately 90%.

## Evaluation of the remaining bone height (RBH)

| Approximal surfaces | Remaining bone height (%) |
|---|---|
| 18D | 90% |
| 18M | 90% |
| 17D | 90% |
| 17M | 90% |
| 16D | 90% |
| 16M | 90% |
| 15D | 90% |
| 15M | 90% |
| 14D | 90% |
| 14M | 90% |
| 13D | 90% |
| 13M | 90% |
| 12D | 90% |
| 12M | 90% |
| 11D | 90% |
| 11M | 90% |
| 21M | 90% |
| 21D | 90% |
| 22M | 90% |
| 22D | 90% |
| 23M | 90% |
| 23D | 90% |
| 24M | 90% |
| 24D | 90% |
| 25M | 90% |
| 25D | 90% |
| 26M | 90% |
| 26D | 90% |
| 27M | 90% |
| 27D | 90% |
| 28M | 90% |
| 28D | 90% |
| 38D | 90% |
| 38M | 90% |
| 37D | 90% |
| 37M | 90% |
| 36D | 90% |
| 36M | 90% |
| 35D | 90% |
| 35M | 90% |
| 34D | 90% |
| 34M | 90% |
| 33D | 90% |
| 33M | 90% |
| 32D | 90% |
| 32M | 90% |

| | |
|---|---|
| 31D | 90% |
| 31M | 90% |
| 41M | 90% |
| 41D | 90% |
| 42M | 90% |
| 42D | 90% |
| 43M | 90% |
| 43D | 90% |
| 44M | 90% |
| 44D | 90% |
| 45M | 90% |
| 45D | 90% |
| 46M | 90% |
| 46D | 90% |
| 47M | 90% |
| 47D | 90% |
| 48M | 90% |
| 48D | 90% |
