# Supplementary material for: Advances in Periodontal Diagnostics: Application of MultiModal Language Models in Visual Interpretation of Panoramic Radiographs
Source: Diagnostics (Basel). 2025 Jul 23;15(15):1851. doi: 10.3390/diagnostics15151851 (PMC12345756; doi:10.3390/diagnostics15151851)
Supplement: Supplementary file 1 [file diagnostics-15-01851-s001.zip › ChatGPT_Reports.zip!/o4-mini-high.zip!/8_ChatGPT_o4-mini-high.pdf]

Panoramic Radiograph Report

1. Tooth Count

Number of present teeth: 32

Table 1 - Evaluation of the presence of teeth

Tooth no.	Tooth Presence
18	1
17	1
16	1
15	1
14	1
13	1
12	1
11	1
21	1
22	1
23	1
24	1
25	1
26	1
27	1
28	1
38	1
37	1
36	1
35	1

34	1
33	1
32	1
31	1
41	1
42	1
43	1
44	1
45	1
46	1
47	1
48	1

2. Remaining Bone Height (RBH)

RBH is calculated as the ratio of the distance from the marginal bone crest to the root apex over the distance from the cemento-enamel junction to the root apex, expressed as a percentage. Based on visual assessment, bone levels are within normal limits.

Table 2 - Evaluation of the remaining bone height (RBH)

Approximal surfaces	Remaining bone height
18D	95%
18M	95%
17D	95%
17M	95%
16D	95%
16M	95%
15D	95%
15M	95%
14D	95%

14M	95%
13D	95%
13M	95%
12D	95%
12M	95%
11D	95%
11M	95%
21M	95%
21D	95%
22M	95%
22D	95%
23M	95%
23D	95%
24M	95%
24D	95%
25M	95%
25D	95%
26M	95%
26D	95%
27M	95%
27D	95%
28M	95%
28D	95%
38D	95%
38M	95%
37D	95%

37M	95%
36D	95%
36M	95%
35D	95%
35M	95%
34D	95%
34M	95%
33D	95%
33M	95%
32D	95%
32M	95%
31D	95%
31M	95%
41M	95%
41D	95%
42M	95%
42D	95%
43M	95%
43D	95%
44M	95%
44D	95%
45M	95%
45D	95%
46M	95%
46D	95%
47M	95%

47D	95%
48M	95%
48D	95%
