# Supplementary material for: Advances in Periodontal Diagnostics: Application of MultiModal Language Models in Visual Interpretation of Panoramic Radiographs
Source: Diagnostics (Basel). 2025 Jul 23;15(15):1851. doi: 10.3390/diagnostics15151851 (PMC12345756; doi:10.3390/diagnostics15151851)
Supplement: Supplementary file 1 [file diagnostics-15-01851-s001.zip › ChatGPT_Reports.zip!/o4-mini-high.zip!/9_ChatGPT_o4-mini-high.pdf]

Panoramic Radiograph Report

Total number of present teeth: 14

Table 1. Evaluation of the presence of teeth

Tooth no.	Tooth Presence
18	0
17	0
16	0
15	0
14	0
13	1
12	1
11	1
21	1
22	1
23	1
24	0
25	0
26	0
27	0
28	0
38	0
37	0
36	0
35	0

34	1
33	1
32	1
31	1
41	1
42	1
43	1
44	1
45	0
46	0
47	0
48	0

Table 2. Evaluation of the remaining bone height (RBH)

Approximal surfaces	Remaining bone height
18D	X
18M	X
17D	X
17M	X
16D	X
16M	X
15D	X
15M	X
14D	X
14M	X
13D	80%
13M	80%
12D	80%
12M	80%
11D	80%
11M	80%
21M	80%
21D	80%
22M	80%
22D	80%
23M	80%
23D	80%
24M	X
24D	X

25M	X
25D	X
26M	X
26D	X
27M	X
27D	X
28M	X
28D	X
38D	X
38M	X
37D	X
37M	X
36D	X
36M	X
35D	X
35M	X
34D	80%
34M	80%
33D	80%
33M	80%
32D	80%
32M	80%
31D	80%
31M	80%
41M	80%
41D	80%

42M	80%
42D	80%
43M	80%
43D	80%
44M	80%
44D	80%
45M	X
45D	X
46M	X
46D	X
47M	X
47D	X
48M	X
48D	X
